# Mechanisms of CPT1C-Dependent AMPAR Trafficking Enhancement

**DOI:** 10.3389/fnmol.2018.00275

**Published:** 2018-08-08

**Authors:** Esther Gratacòs-Batlle, Mireia Olivella, Nuria Sánchez-Fernández, Natalia Yefimenko, Federico Miguez-Cabello, Rut Fadó, Núria Casals, Xavier Gasull, Santiago Ambrosio, David Soto

**Affiliations:** ^1^Laboratori de Neurofisiologia, Departament de Biomedicina, Facultat de Medicina i Ciències de la Salut, Institut de Neurociències, Universitat de Barcelona, Barcelona, Spain; ^2^Institut d’Investigacions Biomèdiques August Pi i Sunyer (IDIBAPS), Barcelona, Spain; ^3^Grup de Recerca en Bioinformàtica i Estadística Mèdica, Universitat de Vic, Barcelona, Spain; ^4^Laboratori de Neurobiologia, Department de Patologia i Terapèutica Experimental, Facultat de Medicina i Ciències de la Salut, Campus Universitari de Bellvitge, Universitat de Barcelona, Barcelona, Spain; ^5^Department de Ciències Bàsiques, Facultat de Medicina i Ciències de la Salut, Universitat Internacional de Catalunya, Barcelona, Spain; ^6^Centro de Investigación Biomédica en Red de Fisiopatología de la Obesidad y la Nutrición (CIBEROBN), Instituto de Salud Carlos III, Barcelona, Spain; ^7^Unitat de Bioquímica, Departament de Ciències Fisiològiques, Facultat de Medicina i Ciències de la Salut, Campus Universitari de Bellvitge, Universitat de Barcelona, Barcelona, Spain; ^8^Institut d’Investigacions Biomèdiques de Bellvitge (IDIBELL), Barcelona, Spain

**Keywords:** AMPARs, CPT1C, surface expression, current density, palmitoylation, AMPAR trafficking, hippocampal pyramidal neurons

## Abstract

In neurons, AMPA receptor (AMPAR) function depends essentially on their constituent components:the ion channel forming subunits and ion channel associated proteins. On the other hand, AMPAR trafficking is tightly regulated by a vast number of intracellular neuronal proteins that bind to AMPAR subunits. It has been recently shown that the interaction between the GluA1 subunit of AMPARs and carnitine palmitoyltransferase 1C (CPT1C), a novel protein partner of AMPARs, is important in modulating surface expression of these ionotropic glutamate receptors. Indeed, synaptic transmission in CPT1C knockout (KO) mice is diminished supporting a positive trafficking role for that protein. However, the molecular mechanisms of such modulation remain unknown although a putative role of CPT1C in depalmitoylating GluA1 has been hypothesized. Here, we explore that possibility and show that CPT1C effect on AMPARs is likely due to changes in the palmitoylation state of GluA1. Based on *in silico* analysis, Ser 252, His 470 and Asp 474 are predicted to be the catalytic triad responsible for CPT1C palmitoyl thioesterase (PTE) activity. When these residues are mutated or when PTE activity is inhibited, the CPT1C effect on AMPAR trafficking is abolished, validating the CPT1C catalytic triad as being responsible for PTE activity on AMPAR. Moreover, the histidine residue (His 470) of CPT1C is crucial for the increase in GluA1 surface expression in neurons and the H470A mutation impairs the depalmitoylating catalytic activity of CPT1C. Finally, we show that CPT1C effect seems to be specific for this CPT1 isoform and it takes place solely at endoplasmic reticulum (ER). This work adds another facet to the impressive degree of molecular mechanisms regulating AMPAR physiology.

## Introduction

Amongst ionotropic glutamate receptors, α-amino-3-hydroxy-5-methyl-4-isoxazolepropionic acid receptors (AMPARs) are considered the “work-horses” of fast excitatory neurotransmission in the brain since they mediate nearly 90% of synaptic transmission. AMPARs are homo- or heterotetrameric structures, in which four different types of subunits (GluA1, 2, 3 and 4) form the basic core of the receptor by making a cationic pore mainly permeable to Na^+^ and K^+^ ions but also Ca^2+^ ions in GluA2-lacking AMPARs (Traynelis et al., 2010). Essentially, the presence or absence within the receptor of a given AMPAR subunit is an important determinant of its properties, which will finally translate into differential integration of the signals at postsynaptic sites. Additionally, AMPAR function in neurons is finely regulated by a noticeable array of interacting proteins, which determine their biophysical properties and govern their intracellular trafficking, exocytosis, endocytosis and synaptic targeting. Actually, in the last decade, the AMPAR field has experienced a fascinating step forward due to the discovery of AMPAR auxiliary subunits, which are important modulators of AMPAR function (Yan and Tomita, 2012; Greger et al., 2017). There are several transmembrane proteins belonging to different families that have been described to command AMPAR function: transmembrane AMPAR regulatory proteins (TARPs), Cornichon homologs (CNIHs), Cystine-knot AMPAR modulating proteins (CKAMPs) and the more recently discovered GSG1L protein (Straub and Tomita, 2012; Farrow et al., 2015; McGee et al., 2015).

Some recent proteomic studies performed in brain extracts confirmed the physical interaction of GluA subunits with these previously mentioned auxiliary proteins and interestingly revealed several new partners of AMPARs (Schwenk et al., 2012, 2014). Amongst the newly identified proteins, carnitine palmitoyltransferase 1C (CPT1C) was shown later to be important in AMPAR function (Gratacòs-Batlle et al., 2015; Fadó et al., 2015). Specifically, GluA1-containing AMPARs were trafficked more efficiently to the plasma membrane by CPT1C (Gratacòs-Batlle et al., 2015) and the absence of this protein in CPT1C knockout (KO) animals was translated into a decrease in miniature excitatory postsynaptic currents (mEPSCs) in hippocampal neurons (HPNs) in culture (Fadó et al., 2015). Furthermore, EPSCs in mossy cells from hippocampal slices were diminished after CPT1C knockdown with short hairpin RNAs (Brechet et al., 2017). Another member of the CPT1 family, CPT1A, has been described not to increase GluA1-mediated currents (Gratacòs-Batlle et al., 2015). This suggests that the mechanism of traffic enhancement is unique to CPT1C. CPT1C also seems to be involved in the synthesis of GluA1 acting as a regulator of AMPAR levels (Fadó et al., 2015) and it has been recently published that CPT1C forms part of specific TARPless AMPAR complexes present in the endoplasmic reticulum (ER) where it has been suggested to prepare—together with Ferric Chelate Reductase 1 like (FRRS1L) as a priming complex—AMPARs for further assembly with TARPs and CNIHs to allow receptors to exit the ER (Brechet et al., 2017). Despite the evident relevance of CPT1C in AMPAR trafficking, the molecular events leading to an enhancement of GluA1 surface expression have not been unraveled.

CPT1C, together with CPT1A and CPT1B belongs to the family of carnitine long-chain acyltransferases (Casals et al., 2016; CPT1s). While CPT1A and CPT1B are localized in the outer membrane of mitochondria (Broadway et al., 2003), CPT1C is located in the ER of neurons (Sierra et al., 2008; Carrasco et al., 2012), which suggests a functional difference between CPT1C and the other members of the family. CPT1A and CPT1B are composed of a small N-terminal domain and a large catalytic C-terminal domain, separated by two transmembrane domains and a short connecting loop with both N and C terminal regions exposed to the cytosolic side of the mitochondrial membrane (Fraser et al., 1997). No crystal structures are available for CPT1 enzymes, but there is for other members of the acyltransferase family (Wu et al., 2003). CPT1s catalyze the exchange of acyl groups between CoA and carnitine to facilitate the transport of long chain fatty acids from the cytoplasm to the mitochondria for β-oxidation (McGarry and Brown, 1997). CPT1A and CPT1B have a catalytic histidine 473, which turns out to be crucial for CPT1s enzymatic reaction (Morillas et al., 2001). This residue is also conserved in CPT1C (His 470) and despite the fact that CPT1C binds to palmitoyl-CoA as CPT1A and CPT1B, its CPT1 catalytic activity is 100-fold lower than other CPT1s (Sierra et al., 2008). This low catalytic activity has been long considered residual but could indeed indicate that CPT1C presents differences in its catalytic function compared to other CPT1s.

The palmitoylation state of AMPARs is important for their delivery to plasma membrane in neurons (Hayashi et al., 2005). Specifically, depalmitoylation of cysteine 585 in GluA1 favors trafficking to the plasma membrane. We previously demonstrated that CPT1C enhancement of GluA1-containing AMPARs surface expression is dependent on the palmitoylable residue cysteine 585 (Gratacòs-Batlle et al., 2015) suggesting that changes in the palmitoylation state of GluA1 mediated by CPT1C might be responsible for this effect. However, no depalmitoylation activity has been demonstrated for CPT1C. Indeed, one of the best studied depalmitoylating enzymes is the acyl protein thioesterase 1 (APT1), a cytosolic enzyme that catalyzes depalmitoylation of membrane anchored proteins (Zeidman et al., 2009; Salaun et al., 2010) through the catalytic Ser 119, Asp 174 and His 208 triad in *human* APT1 sequence (Devedjiev et al., 2000; Wang et al., 1997).

In the present study, we have focused on unraveling the molecular mechanism underlying CPT1C-mediated AMPAR modulation. Combining *in silico* and experimental approaches we have identified Ser 252-His 470-Asp 474 as the catalytic triad in CPT1C involved in depalmitoylating activity. Mutagenesis studies of these key residues abolished CPT1C effect on AMPAR trafficking. In addition, inhibition of CPT1C activity by Palmostatin-B (PB), an inhibitor of palmitoyl thioesterase (PTE) activity in APT1 (Dekker et al., 2010), impedes CPT1C modulation of GluA1-mediated AMPAR currents. Thus, we propose that CPT1C modulates AMPAR trafficking through depalmitoylation of GluA1.

## Materials and Methods

### CPT1C Computational Molecular Model

#### *In Silico* CPT1C Molecular Model

An initial homology model was constructed for the catalytic domain of *human* CPT1C using the coordinates of the determined X-ray crystal structure of *human* carnitine acetyltransferase (PDB ID: 1NM8, 1.8 Å of resolution, 30% of sequence identity; Wu et al., 2003). Modeller 9.12 (Sali and Blundell, 1993) was used to model the non-determined regions. The side chain conformations for non-conserved residues were positioned according to Scwrl 4 (Krivov et al., 2009). The protein was embedded in a tip3p water box. The initial system was energy minimized, subjected to 10 ns of molecular dynamics equilibration and finally to a production stage extending to 150 ns. All the simulations were performed with GROMACS 5.0 simulation package (Berendsen et al., 1995).

The relative disposition of residues Ser114, Asp169 and His203 that constitute the catalytic triad in *human* APT1 (PDB ID: 1FJ2, 1.5 Å; Devedjiev et al., 2000) were used to identify the putative serine and aspartate residues that together with His 470 constitute the catalytic triad in *human* CPT1C. Structural superimposition of *human* CPT1C molecular model to *human* APT1 structure with PyMOL (PyMOL) identified Ser 252 and Asp 474 as the two residues that together with His 470 constitute the catalytic triad.

#### CPT1C—CoA—Carnitine—Palmitate

The position of carnitine and CoA in the binding pocket was obtained by structurally superimposing the structure of *murine* carnitine acetyl-transferase in complex with acetyl-CoA and carnitine (PDB ID: 2H3U, 1.9 Å) to *human* CPT1C molecular model with PYMOL (PyMOL) resulting in 0.445 Å of root mean square deviation (RMSD). In order to obtain the position of palmitate, the structure of *rat* carnitine palmitoyltransferase II (PDB ID: 4EP9, 2.03 Å) was structurally superimposed to a CPT1C molecular model with PYMOL (PyMOL), resulting in 1.106 Å of RMSD. The final structure of CPT1C in complex with CoA, carnitine and palmitate was energy minimized.

### Animals and Housing

C57BL/6J Wild-type (WT) and CPT1C KO mice (MGI database ID: 5432790) were provided by the laboratory of Dr. Núria Casals (Universitat Internacional de Catalunya) and were obtained as described in Carrasco et al. (2012). Animals were housed in cages with free access to food and water and were maintained under controlled day–night cycles in accordance with the NIH Guide for the Care and Use of Laboratory Animals, the European Union Directive (2010/63/EU), and the Spanish regulations on the protection of animals used for research, following a protocol approved and supervised by the CEEA-UB (Ethical Committee for Animal Research) from University of Barcelona with the license number OB117/16, of which DS is the responsible researcher.

### Cell Lines Culture and Transfection

HEK293-AD, COS-7 and tsA201 cell lines were used in this study. tsA201—or HEK293T—are HEK293 cells that constitutively express the SV40 large T antigen to allow plasmid replication using the SV40 origin and hence to produce high levels of recombinant proteins (Sigma catalog 85120602). Cells were maintained as described in Gratacòs-Batlle et al. (2015). Cells were transiently co-transfected with 5.4 μg total cDNA (for Co-IP) and 0.6 μg total cDNA (for immunofluorescence, IF and electrophysiology) using PEI transfection reagent (1 mg/ml) in a 3:1 ratio (PEI:DNA). In all transfections the DNA ratio used was 1:2 (GluA:CPT1C). Media was replaced 3–5 h after transfection with fresh media containing 2,3-dioxo-6-nitro-1,2,3,4-tetrahydrobenzo [f]quinoxaline-7-sulfonamide at 50 μM (NBQX; Tocris-ABCam) to prevent excessive AMPAR-mediated toxicity. For electrophysiology experiments, cells were re-plated on coated glass coverslips to allow optimal density. All experiments were performed 48 h after transfection.

### Hippocampal Neuronal Cultures and Transfection

Hippocampal neuronal cultures were performed from P0 to P2 (aged 0–2 days) WT or CPT1C-KO C57BL/6J mice in accordance with Catalan animal procedures (Decret 214/97; Generalitat de Catalunya) as described in Coombs and Soto (2016). Briefly, hippocampi were isolated and maintained in precooled Hank’s Balanced Salt Solution (HBSS, Gibco) supplemented with 0.45% glucose. Hippocampi were chopped and chemically digested with Trypsin (Type XI; Sigma). After two washes of the tissue with pre-warmed HBSS, a mechanical digestion was performed in plating media by passing hippocampal tissue through decreasing tip diameter sigmacoated glass Pasteur pipettes to achieve a single cell suspension. The dissociated cell suspension was carefully placed on top of a pre-warmed ovomucoid-BSA solution (10 mg/ml in HBSS for each) and centrifuged (1,000× *g*) during 10 min. The resulting pellet was resuspended in platting media (Coombs and Soto) and seeded on poly-D-Lysine/laminin-coated 12 mm ∅ coverslips (VWR International) into 24-well plates at a density of 100,000–150,000 cells/well. AraC (Sigma) was added at 2 days *in vitro* (d.i.v.) at a concentration of 5 μM. 2/3 of fresh maintenance media containing AraC was changed every 4–5 days.

Neuronal cultures were transiently transfected with 0.8 μg total cDNA for both IF and electrophysiology using Lipofectamine^®^ 2000 (Invitrogen) following manufacturers indications. Media was replaced 4 h after transfection with conditioned medium. All experiments were performed between 48 h and 72 h after transfection.

### Expression Constructs

To obtain CPT1C-EGFP (referred hereafter as CPT1C-GFP or CPT1C) cDNAs with mutations in the putative thioesterase catalytic residues, we used site-directed mutagenesis to change specific base pairs. Primers containing the desired mutation were designed and obtained at Sigma-Aldrich.

CPT1C(H470A) and CPT1C-(S252A, H470A, D474A) mutant cDNAs resulted from changing codons: CAC to GCC, GAC to GCC and AGC to GCC respectively and sequentially for the triple mutant. All changes produce an alanine instead of the potentially active residue as has been performed previously for mutating the catalytic triad of many thioesterases (Devedjiev et al., 2000; Tian et al., 2012; Yokoi et al., 2016). The primers used for introducing the mutations were the following: *CPT1C-S252A*: ggctcgctggttaatGCcacctactacatgatgg *CPT1C-H470A*: ctcagcgtggagGCctcatgggctgactgc *CPT1C-D474A*: ggcctcatgggctgCctgccctgtcgcggg

All constructs were fully sequenced to verify sequence integrity.

AMPAR subunit cDNAs were a gift from S. Heinemann (Salk Institute, La Jolla, CA, USA) and P. Seeburg (Max Planck Institute, Heidelberg, Germany). DHHC-3/GODZ expression vector was a gift from Luke Chamberlain (Strathclyde University). pDs-Red-ER-KDEL and pGFP-Sec61B constructs were a gift from Juan Pablo Muñoz (IRB, Barcelona). The characteristics of CPT1C and CPT1A plasmid vectors were described in Gratacòs-Batlle et al. (2015). Plasmid constructs for the expression of chimeric proteins *C*-CPT1A and *A*-CPT1C where obtained as described in Sierra et al. (2008). All plasmid vectors are under the control of CMV promoter.

### Coimmunoprecipitation

#### Coimmunoprecipitation From tsA201 Cells

Forty-eight hours after transfection, tsA201 cells grown in T-25 flasks were washed twice with room temperature (RT) PBS and scraped with ice-cold lysis buffer (1% Triton TX-100, 50 mM Tris-HCl pH 8, 150 mM NaCl, Protease Inhibitor Cocktail (Roche) and PMSF). All subsequent steps were performed at 4°C. Cells were lysed with a 30 G syringe six times and membranes were solubilized during 30 min in an orbital agitator. Insoluble material was pelleted at 16,000× *g* for 30 min. Protein concentration in the supernatant was quantified by the BCA method and 0.4–1 mg of protein was incubated overnight with 2–4 μg of anti-GluA1-NT (MerckMillipore) or with anti-GFP antibody (rabbit serum from Life Technologies) with orbital agitation. Antibody-protein complexes were pulled down by incubating with 80–100 μl of Protein-A sepharose beads (Sigma) for 2–3 h. Precipitated complexes were washed with lysis buffer three times and eluted with 2× SB/5 mM DTT sample buffer, heated 10 min at 76°C and separated by SDS/PAGE. Before adding the antibodies, 10% of total protein was removed as input samples and boiled at 75°C for 10 min in 2× SB/5 mM DTT.

#### Coimmunoprecipitation From Mouse Brain

Hippocampi, cerebellums and frontal cortices were obtained from C57BL/6J WT and CPT1C KO mice of 1 or 2 months of age. After dissection, they were sonicated in cold Tris·HCl pH 7.4 (with Protease Inhibitor Cocktail (Roche) and PMSF) with a Broanson Sonifier 150 (5 watts). All subsequent steps were performed at 4°C. The homogenate was centrifuged at 16, 000 xg for 30 min at 4°C. The pellet was resuspended by pipetting up and down in ice-cold lysis buffer (1% IGEPAL, 50 mM Tris-HCl pH 7.5, 150 mM NaCl, 10% Glycerol, Protease Inhibitor Cocktail (Roche) and PMSF) and solubilized during 30 min in an orbital agitator. Insoluble material was pelleted at 16,000× *g* for 30 min. Protein concentration in the supernatant was quantified by the BCA method and 1.5 mg of protein were incubated overnight with 2 μg of anti-GluA1 antibody with orbital agitation. Antibody-protein complexes and inputs were obtained as described in coimmunoprecipitation from tsA201 cells.

### Immunoblotting

Samples were separated in 8% mini-protean SDS/PAGE gels, transferred using Trans-Blot Turbo transfer system on PVDF membranes (all from BioRad). Membranes were blocked in TBS with 0.1% Tween 20 (TBS-T) containing 3% (wt/vol) BSA. Primary antibodies used to detect different proteins were the same described for immunoprecipitations and anti-CPT1C (RRID:AB_2636893, Sierra et al., 2008; Pozo et al., 2017). Peroxidase-conjugated goat anti-mouse or goat anti-rabbit secondary antibodies diluted in blocking solution, were detected by using WesternBright ECL (Advansta) and exposed to light-sensitive film (Amersham Hyperfilm).

### Immunofluorescence

IF was performed in tsA201 cells and neuronal cultures grown on lysine treated coverslips (plus laminin for neurons), 48 h after transfections. Washes were performed by immersion of the coverslips in PBS with Calcium and Magnesium (PBS-CM) or PBS-G (20 mM Glycine in PBS-CM). Composition of solutions were as follows: fixation solution (4% PFA in PBS), permeabilization solution (0.1% Triton X-100 in PBS-G −0.3% for neurons), blocking solution (10% NGS, 2% BSA, 0.1% Triton X-100 in PBS-G), antibody incubation solution (4% normal goat serum and 0.1% BSA in PBS-G) and triton-antibody solution (antibody incubation solution containing 0.1% Triton X-100). Incubations with antibodies were performed in a humid chamber at 37°C for 1 h.

Surface staining of AMPARs was achieved by labeling live cells with the mouse anti-GluA1-NT (from Merck Millipore) in a 1:500 solution in DMEM:F12, for 10 min at 37°C for cell lines or in a 1:500 solution in Neurobasal/B27, for 1 h at 37°C for neurons. After six washes in RT PBS-CM, cells were fixed for 15 min at RT and incubated with goat anti-mouse Alexafluor 555 (Molecular Probes) diluted 1:500 in antibody incubation solution. After several washes in PBS-CM, cells were fixed again to preserve the binding of the first secondary antibody. Cells were subsequently permeabilized for 5–10 min and blocked for 30 min. Next, and in order to determine the intracellular expression of AMPARs in each cell, GluA1 were labeled, incubating the coverslips with the same mouse anti-GluA1-NT antibody at 1:500 (in triton-antibody incubation solution). Following washes in PBS-CM, cells were incubated with goat anti-mouse Alexafluor 647 (Molecular Probes) at 1:500 (in triton-antibody incubation solution). Coverslips were then washed and mounted with Mowiol (Calbiochem).

For co-localization of *A*-CPT1C or *C*-CPT1A, the following method was used: COS-7 cells were transfected with the different CPT1-GFP tagged plasmid constructs with or without an ER marker (200 ng of RE-KDEL-dsRed tagged). For co-localization with a mitochondrial marker, transfected cells were incubated with MitoTracker (Thermo Fisher Scientific) at 25 nM during 20 min at 37°C. Cells were washed in PBS, fixed in 4% PFA for 15 min and mounted in Fluoromount.

For co-localization of CPT1C with a COPII marker (mouse Sec31A, BD Biosciences), COS-7 cells were transfected with 200 ng of CPT1C-GFP or with pGFP-Sec61B plasmid. Forty-eight hours after transfection cells were fixed for 15 min in 4% PFA, washed and IF against Sec31A (1:100) with Alexafluor 555 antimouse at 1:500 (Molecular Probes) was performed.

### Confocal Imaging and Immunofluorescence Quantification

Confocal images were acquired with a Leica TCS SP5 laser scanning confocal microscope (Leica Microsystems Heidelberg GmbH, Manheim, Germany) equipped with a DMI6000 inverted microscope, blue diode (405 nm), Argon (458/476/488/496/514), diode pumped solid state (561 nm) and HeNe (594/633 nm) lasers and a PLAN APO 63× oil (NA 1.4) immersion objective lens. DAPI, GFP, Alexa Fluor 555 and Alexa Fluor 647 images were acquired sequentially using 405, 458, 488, 561 and 633 laser lines, AOBS (Acoustic Optical Beam Splitter) as beam splitter and emission detection ranges 415–480, 500–550 nm, 571–625 and 643–680 nm respectively and the confocal pinhole set at 1 Airy units. Images were acquired at 600 Hz in a 1024 × 1024 pixels format, zoom at 1 and pixel size of 240.3 × 240.3 nm, for imaging tsA201 and zoom at 3 with a pixel size of 80.2 × 80.2 nm for neurons.

IFs quantification was performed as described in Gratacòs-Batlle et al. (2015) using ImageJ (NIH). A set of three different IF images for each condition were performed and 30–80 cells from each condition were analyzed for each IF. For cortical and HPNs images were taken from 3 to 5 different cultures. Quantification of co-localization was performed using the Manders’ Overlap coefficient (MOC) calculated in ImageJ via the JACoP plugin from images of single cells. This coefficient ranges between 1 and zero with 1 being high co-localization, zero being low.

### Electrophysiology: Whole-Cell Recordings on tsA201 Cells and Hippocampal Neurons

Whole-cell recordings were performed from isolated transfected cells visualized with an inverted epifluorescence microscope (Axio-Vert.A1; Zeiss). Thick-walled electrodes were fabricated from borosilicate glass (1.50 mm O.D., 1.16 mm I.D., Harvard Apparatus) pulled with a P-97 horizontal puller (Sutter Instruments) with a final electrode resistance of 3–5 MΩ. In tsA201 transfected cells, macroscopic currents were activated from GFP-positive cells by a bath application of 1 mM glutamate plus 25 μM cyclothiazide to prevent receptor desensitization and were recorded by applying a voltage ramp protocol from –80 mV to +80 mV at a rate of 160 mV/s) as previously described in Gratacòs-Batlle et al. (2015). In pyramidal HPNs currents were activated by a 20 s rapid piezo-driven application of 20 μM AMPA +10 μM cyclothiazide. In both types of recordings, to avoid errors due to differences in cell surface area, the responses were expressed as current density (–pA/pF; current at –80 mV (tsA201 cells) or –60 mV (HPNs) divided by input capacitance). Currents were recorded with Axopatch 200B amplifier, filtered at 2 kHz and digitized at 5 kHz using Digidata 1440A interface with pClamp 10.2 software (Molecular Devices Corporation). For tsA201 cell recordings the “extracellular” solution contained (in mM): 145 NaCl, 2.5 KCl, 1 CaCl_2_, 1 MgCl_2_, 10 glucose and 10 HEPES (pH to 7.42 with NaOH). The “intracellular” solution contained (in mM): 145 CsCl, 2.5 NaCl, 1 Cs-EGTA, 4 MgATP and 10 HEPES (pH = 7.2 with CsOH). For HPNs recordings the “extracellular” solution contained (in mM): 140 NaCl, 3.5 KCl, 1.8 CaCl_2_, 0.8 MgCl_2_, 20 glucose and 10 HEPES (pH to 7.42 with NaOH). The “intracellular” solution contained (in mM): 116 K-gluconate, 6 KCl, 8 NaCl, 0.2 EGTA, 2 MgATP, 0.3 NaGTP and 10 HEPES (pH = 7.2 with KOH). TTX 1 μM was added to block synaptic transmission. The specific blockers of NMDARs and GABA_A_Rs, APV 25 μM and picrotoxin 100 μM were present to minimize background noise due to spontaneous release of neurotransmitters. All recordings were obtained for at least three different hippocampal neuronal cultures. Spermine tetrahydrochloride (Sigma Aldrich) was added to intracellular solution at 100 μM in all electrophysiology experiments.

### Acyl-Biotin Exchange Assay (ABE)

Detection of palmitoylation levels of GluA1 subunits was performed exactly as described in Brigidi and Bamji (2013). HEK293-AD cells stably expressing GluA1 were transfected with 2 μg of GODZ (pEF-BOS-HA-DHHC3) and 4 μg of GFP, CPT1C-GFP or CPT1C(H470A)-GFP cDNA’s. Forty-eight hours after transfection, cells were washed twice with RT PBS with Calcium and Magnesium and scraped in ice-cold lysis buffer (LB: 1% IGEPAL, 50 mM Tris-HCl pH 7.5, 150 mM NaCl, 10% Glycerol, Protease Inhibitor Cocktail (Roche) and PMSF) containing 50 mM N-ethylmaleimide NEM (Sigma). Then, cells were lysed with a 30 G syringe six times and incubated 20 min with orbital agitation at 4°C. All steps where performed at 4°C. Lysates were cleared by centrifugation at 16,000× *g* for 30 min and the amount of protein in the supernatant was determined using the BCA method (Thermo Scientific). 750 μg–1.5 mg of protein were used for overnight immunoprecipitation of GluA1 (4 μg of anti-GluA1-NT antibody (Merck Millipore)). Then, protein-antibody complexes were pulled-down with Protein-A sepharose beads (Sigma) preequilibrated with LB/50 mM NEM for 1.5 h. The total immunoprecipitate was then resuspended in LB with 10 mM NEM and was split into two equivalent samples: one sample for specific cleavage and unmasking of the palmitoylated cysteine’s thiol group by 1 M hydroxylamine treatment (+HAM sample) and a second equivalent sample in the absence of HAM to control non-specific incorporation of biotin (−HAM sample). Before performing HAM treatment, samples were washed extensively to remove unbound NEM (one rapid wash with Stringent buffer (LB/10 mM NEM and 0.1% SDS) and three washes with LB (pH 7.2)). 1 M HAM solution was prepared in pH 7.2 LB and ±HAM treatment was performed with rotation for 1 h at RT. After one wash in LB pH 6.2, selective labeling of the basal palmitoylated cysteines (which after HAM treatment becomes a cysteine with a free-thiol group) using a thiol-reactive biotinylation reagent, biotin-BMCC (1 μM; Thermo Scientific) in pH 6.2 LB was performed for 1 h at 4°C with orbital agitation in ±HAM samples. Afterwards, the thiol-biotinylated proteins following the acyl-biotin exchange assay (ABE) steps were washed three times in LB (pH 7.5) and resolved by SDS-PAGE and Western Blotting was performed. Membranes were blocked with 3% BSA in TBS-T and incubated with Streptavidin-HRP (Invitrogen; 1:5,000 from a 1 mg/ml stock in 0.3% BSA). After stripping (Thermo Fisher Stripping buffer), the same membrane was incubated with an anti-GluA1-NT antibody (1:2,000) to normalize palmitoylation levels to the amount of immunoprecipitated protein.

Western Bright ECL (Advansta) was applied on blotting membrane for protein detection. Images were captured using Chemidoc Digital Imaging system (BioRad) and analyzed with ImageLab 6.0 software (BioRad).

### Analysis and Statistics

Electrophysiological recordings were analyzed using IGOR Pro (Wavemetrics Inc.) with NeuroMatic (Jason Rothman, UCL). Data are presented in the text as the mean ± SEM from n experiments and in the figures as bar plots of the group mean, with error bars denoting the SEM. Comparisons between two groups were performed using the non-parametric Mann-Whitney U test. Differences were considered significant at *p* < 0.05. Statistical analysis was performed using GraphPad Prism version 5.0d for Mac OS X (GraphPad Software, San Diego, CA, USA^1^).

## Results

### GluA1 and CPT1C Coimmunoprecipitate in Different Brain Areas

**Figure 1:**
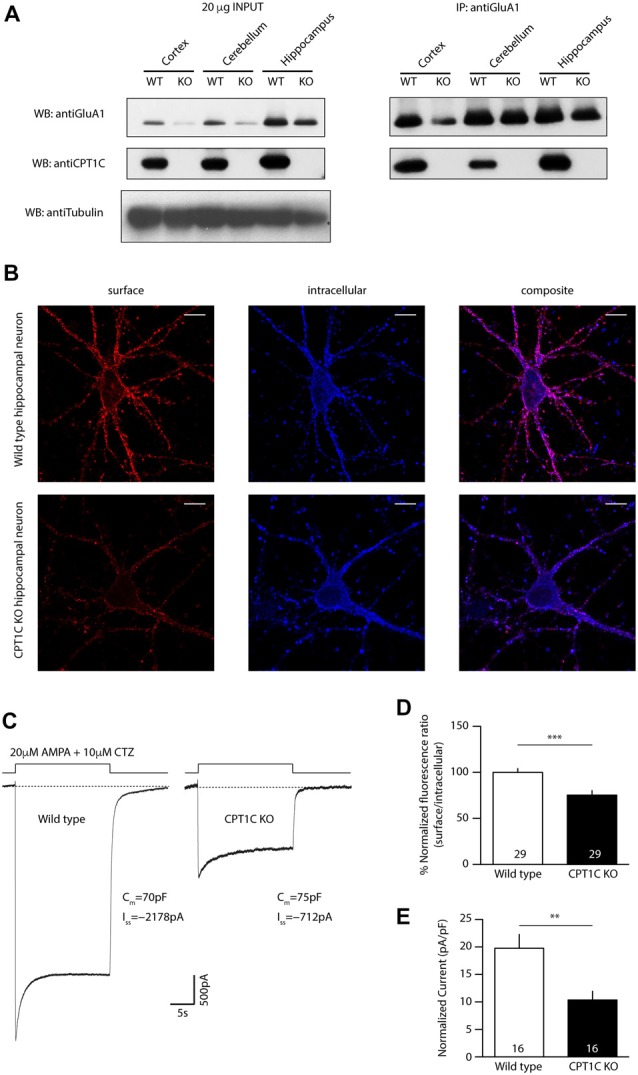
Carnitinepalmitoyltransferase 1C (CPT1C) absence in knockout (KO) animals translates into a lower AMPAR expression at neuronal surface. **(A)** Coimmunoprecipitation of GluA1 and CPT1C from cortex, cerebellum and hippocampal tissue of wild type (WT) and CPT1C KO showing GluA1-CPT1C interaction in all tested brain areas. **(B)** Representative single confocal images of immunofluorescence (IF) of 11 days *in vitro* (DIV) hippocampal pyramidal neurons in culture from WT (upper images) and CPT1C KO (lower images) animals. Surface GluA1 (Alexafluor 555; red signal) and intracellular GluA1 (Alexafluor 647; blue signal) is shown. **(C)** Examples of somatic currents evoked by 20 μM AMPA +10 μM cyclothiazide during a 20 s fast application pulse for WT (left trace) and CPT1C KO (right trace) hippocampal pyramidal neurons. Two neurons with similar cell capacitance are shown. **(D)** Quantification of endogenous somatic GluA1 surface to intracellular ratio from pyramidal cells in culture. GluA1 ratio was diminished in neurons from CPT1C KO animals compared with WT (***p = 0.0003; Mann-Whitney-U-test). Numbers in bars indicate the number of neurons analyzed. **(E)** CPT1C KO pyramidal neurons display lower current density—measured at the steady state current (**p = 0.0035; Mann-Whitney-U-test). Numbers in bars denote the number of recorded cells.

CPT1C protein has been shown to play an important role in GluA1-containing AMPAR surface expression including GluA1/GluA2 heteromers (Gratacòs-Batlle et al., 2015). In HPNs GluA1/GluA2 is the dominant AMPAR arrangement (Pellegrini-Giampietro et al., 1994; Gold et al., 1996; Sans et al., 2003) and in CPT1C KO animals the number of AMPARs at hippocampal neuron synapses seems to be reduced (Fadó et al., 2015). Since GluA1 is also importantly expressed in other brain areas, we first decided to study the ability of CPT1C to interact with GluA1 subunits in different brain tissues. Thus, we performed coimmunoprecipitation assays from cortex, cerebellum and hippocampus of WT and CPT1C KO animals (P24–39). Anti-GluA1 antibody clearly pulled down CPT1C from cortex, cerebellum and hippocampus (Figure 1A, right panel). No CPT1C signal was detected from KO animals (WB: anti-CPT1C; KO lanes). To demonstrate that the binding of CPT1C to GluA1 is specific, as a negative control we used neutral IgG to immunoprecipitate from WT homogenates. By doing so, we proved that CPT1C is definitively pulled down by GluA1 antibodies in different brain areas (Supplementary Figure S1). Thus, these experiments confirmed that CPT1C interacts with GluA1 in all tested tissues and indicates that CPT1C-mediated regulation of AMPARs might be a common event throughout the CNS.

### AMPARs Surface Content Is Diminished in CPT1C KO Mice

Since animals lacking CPT1C protein show reduced AMPAR-mediated mEPSCs in hippocampal pyramidal cells (Fadó et al., 2015), it is likely that somatic AMPAR content might be altered as well in these neurons due to a lack of optimal trafficking. Thus, we decided to study whether extrasynaptic somatic AMPAR content was altered in CPT1C KO animals. First, we performed IF experiments to determine the relative amount of AMPARs on neuronal surface. We immunostained surface GluA1 in primary hippocampal pyramidal neuronal cultures at 11 DIV (Figure 1B; red signal) followed by permeabilization and staining of the intracellular GluA1 pool (Figure 1B; blue signal). We calculated the ratio of the surface expression of GluA1 subunit vs. the intracellular level of GluA1 for every single cell. Figure 1D shows the normalized ratio of surface to intracellular GluA1, where it can be observed that this ratio was decreased in cultures from CPT1C KO animals (100.00 ± 4.13% for WT vs. 75.34 ± 4.86% for CPT1C KO; *p* = 0.0003; *n* = 29 analyzed cells for both conditions from three different cultures). Since CPT1C is also expressed in cortex where it coimmunoprecipitates with GluA1 (Figure 1A), we investigated whether the surface to intracellular ratio of GluA1 could also be altered in cortical neurons in culture. IF experiments were carried out with the same outcome than in HPNs (100.00 ± 5.36% for WT cortical neurons vs. 73.41 ± 4.80% for CPT1C KO cortical neurons; *p* = 0.0006; *n* = 25 analyzed cells for both conditions from three different cultures; Supplementary Figure S2).

We next recorded whole-cell currents activated from hippocampal pyramidal neurons in culture (14–15 DIV) by rapid somatic application of 20 μM AMPA plus 10 μM cyclothiazide to minimize AMPAR rapid desensitization. We found that AMPAR-mediated currents were lower in CPT1C KO HPNs compared to WT neurons (19.76 ± 2.51 pA/pF for WT vs. 10.36 ± 1.59 pA/pF for CPT1C KO; *p* = 0.0035; *n* = 16 cells for both conditions from three and four different cultures, respectively; Figures 1C,E). Thus, in addition to synaptic AMPARs (Fadó et al., 2015), somatic content of AMPARs also seems to be decreased in CPT1C deficient animals reinforcing the positive role of CPT1C in AMPAR trafficking.

### Identification of the Catalytic Triad in CPT1C

**Figure 2:**
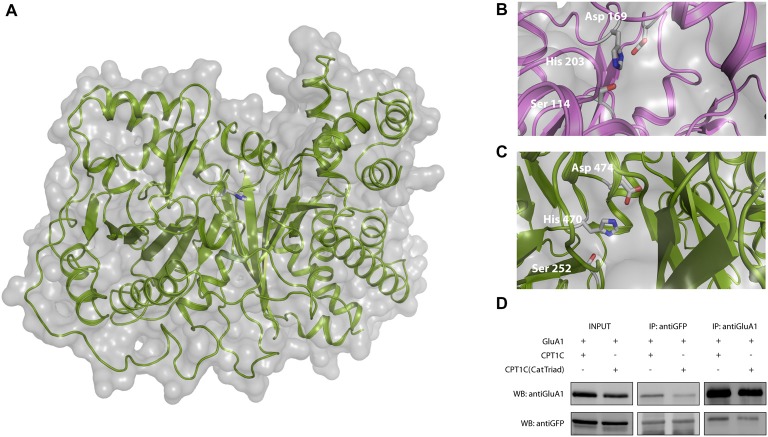
Identification of a putative catalytic triad in CPT1C. **(A)** Molecular model of *human* CPT1C based on the determined structure of *human* carnitine acetyltransferase. Residue His 473, conserved among CPTs (His 470 in CPT1C), is displayed in sticks. **(B)** Structure of acyl protein thioesterase 1 (APT1; PDB ID 1FJ2). Ser 114, His 203 and Asp 169 from the catalytic triad are displayed in sticks. **(C)** CPT1C molecular model. Ser 252, His 470 and Asp 474 putative catalytic residues are displayed in sticks. The putative catalytic residues in CPT1C were identified based on the relative disposition of the catalytic residues in the structure of APT1. **(D)** Coimmunoprecipitation showing that mutated CPT1C(S252A, H470A, D474A)-GFP—CPT1C(CatTriad)—does not affect the interaction with GluA1. Anti-GFP antibodies pull-down GluA1 when expressed together withCPT1C(CatTriad), (middle lanes, upper panel). GluA1 recognizing antibodies pull downCPT1C(CatTriad), (right lanes, lower panel). This experiment was replicated three times.

Previous studies exploring CPT1C function have shown that its canonical CPT1 catalytic activity is much lower than that of CPT1A (Sierra et al., 2008). However, previous investigation has pointed to a role for CPT1C in depalmitoylating AMPARs although attempts to measure depalmitoylation using the Acyl Biotin Exchange assay were not conclusive (Gratacòs-Batlle et al., 2015). Thus, we decided to study this putative depalmitoylating activity through other approaches in order to understand the molecular mechanisms of CPT1C. For this purpose, an *in silico* structural molecular model was constructed for *human* CPT1C based on the crystal structure of *human* carnitine acetyl transferase (Wu et al., 2003). The structural molecular model for *human* CPT1C catalytic domain is shown in Figure 2A, where His 470 is pointing towards the binding pocket. This histidine has been shown to be crucial for CPT1A catalytic activity (Morillas et al., 2001).

We then investigated a possible homology between the structures of CPT1C and APT1, a crystallized depalmitoylating enzyme (Devedjiev et al., 2000). Comparison of these two molecular model structures allowed us to identify two residues that together with His 470 could putatively endow thioesterase activity in CPT1C. Specifically, residues Ser 252, His 470, Asp 474 in CPT1C were located in positions corresponding to residues Ser 114, His 203 and Asp 169 in the *human* APT1 structure (Ser 119, His 208 and Asp 174 and in *human* APT1 sequence; see Figures 2B,C). To validate Ser 252, His 470 and Asp 474 as the catalytic triad, we created a CPT1C-GFP version in which those putative catalytic residues were mutated to alanine, CPT1C(S252A, H470A, D474A)—referred henceforth as CPT1C(CatTriad). In order to explore whether this triple mutation could affect the interaction between both proteins, a coimmunoprecipitation assay was performed and subsequently analyzed by Western blotting (*n* = 3). Figure 2D shows that GFP recognizing antibody could pull down GluA1 when expressed together with CPT1C or CPT1C(CatTriad). Additionally, GluA1-NT antibody could pull down CPT1C and CPT1C(CatTriad) proteins revealing that both proteins still interact regardless of the different mutations introduced in CPT1C and suggesting also that the triple mutation does not affect the native folding of the protein.

**Figure 3:**
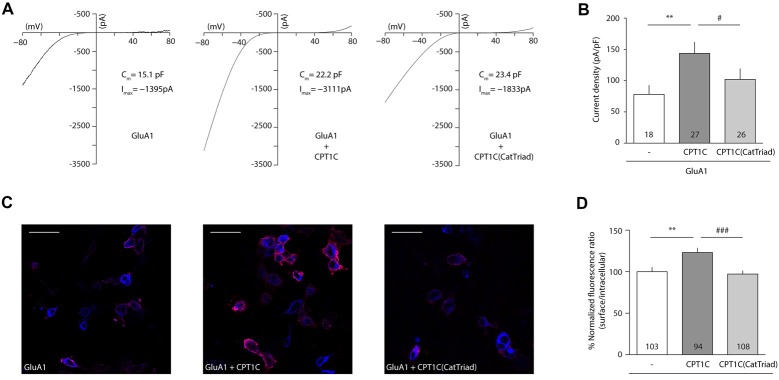
CPT1C possesses a functional catalytic triad. **(A)** Examples of whole-cell currents between –80 mV and +80 mV evoked by 1 mM glutamate plus 25 μM cyclothiazide in tsA201 transfected cells with GluA1 alone (GluA1+GFP), together with CPT1C or CPT1C(CatTriad). Cells with similar cell capacitance are shown. **(B)** Average of current density (–pA/pF) at –80 mV for GluA1 alone and together with CPT1C or CPT1C(CatTriad). CPT1C increased GluA1-mediated currents (**p < 0.01; Mann-Whitney-U-test) while mutation of Ser 252, His 470 and Asp 474 to alanine in CPT1C avoided the enhancement of the currents (^#^*p* < 0.05; Mann-Whitney-U-test). Numbers in bars denote the number of recordings. **(C)** Representative confocal images of tsA201 cells co-transfected with GluA1+GFP (left panel), GluA1+CPT1C (middle panel) or GluA1+CPT1C(CatTriad), (right panel) where surface GluA1 was labeled with anti-GluA1-NT and Alexafluor 555 (red signal) and intracellular GluA1 was labeled with same anti-GluA1-NT primary antibody plus Alexafluor 647 (blue signal). Scale bars: 50 μm. **(D)** Quantification of the GluA1 surface to intracellular ratio normalized to GluA1 and expressed as a percentage. The increment in surface expression due to CPT1C (**p < 0.01; Mann-Whitney-U-test) was abolished when Ser 252, His 470 and Asp 474 were changed to alanine in CPT1C (^###^*p* < 0.001; Mann-Whitney-U-test). Numbers in bars denote the number of quantified cells from three different IF experiments.

Following coimmunoprecipitation assays, electrophysiological whole-cell recordings were performed to assess whether the catalytic triad was important in modulating AMPAR-induced currents. Figure 3A shows typical currents mediated by GluA1 homomeric receptors from –80 mV to +80 mV in the absence of CPT1C or together with CPT1C or CPT1C(CatTriad). GluA1 currents were increased in the presence of CPT1C as previously demonstrated (78.00 ± 14.37 pA/pF for GluA1 alone vs. 143.7 ± 17.79 pA/pF for GluA1+CPT1C; *p* = 0.0027; Mann–Whitney U-test; *n* = 18 and 27, respectively; Figure 3B). Interestingly, there was no statistical difference between current densities in cells expressing GluA1 alone or together with CPT1C(CatTriad; 78.00 ± 14.37 pA/pF vs. 102.1 ± 17.12 pA/pF; *p* = 0.2144; Mann–Whitney U-test; *n* = 18 and 26, respectively; Figure 3B) and current density values for CPT1C(CatTriad) were lower than in CPT1C co-expression (*p* = 0.0134; *n* = 27 and 26 for GluA1+CPT1C and GluA1+CPT1C(CatTriad) respectively). The increase in CPT1C-dependent GluA1-mediated currents has been correlated with an increase in the surface to intracellular ratio of GluA1 subunit (Gratacòs-Batlle et al., 2015). Therefore, we performed IF experiments of GluA1 in the absence or presence of WT or the mutated form of CPT1C (Figure 3C). WT CPT1C increased surface/intracellular GluA1 ratio as previously shown (100.0 ± 5.17% for GluA1alone vs. 123.1 ± 5.43% GluA1+CPT1C; *p* = 0.0048; Mann–Whitney U-test; *n* = 103 and 94 respectively from three different IF; Figure 3D). The analysis of IF experiments showed that the increase in GluA1 ratio content at the cell surface observed with CPT1C was lost in CPT1C(CatTriad; 123.1 ± 5.43% for GluA1+CPT1C vs. 97.15 ± 3.89% GluA1+CPT1C(CatTriad); *p* = 0.0005; Mann–Whitney U-test; *n* = 94 and 108 respectively from three differerent IF; Figure 3D). Indeed, the ratio of GluA1+CPT1C(CatTriad) was not different from GluA1 alone (100.0 ± 5.17% for GluA1 vs. 97.15 ± 3.89% GluA1+CPT1C(CatTriad); *p* = 0.7496; Mann–Whitney U-test; *n* = 103 and 108 respectively from three different IF; Figure 3D). Based on these results we propose that Ser 252, His 470 and Asp 474 constitute the catalytic triad in CPT1C, suggesting that CPT1C has a depalmitoylating activity that is responsible for its effect on GluA1.

### CPT1C H470 Is Crucial in the Enhancement of AMPAR Surface Expression

**Figure 4:**
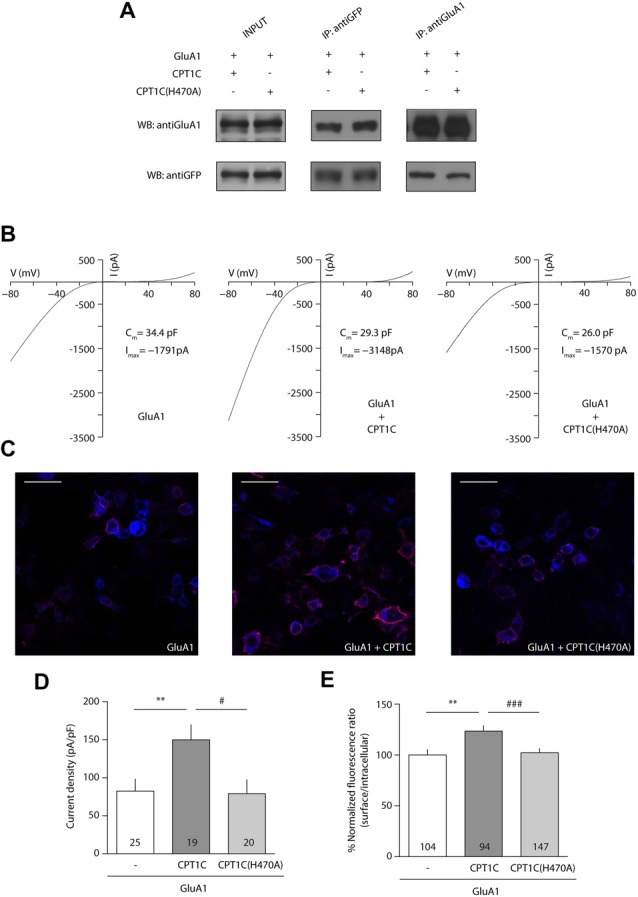
Histidine 470 is crucial in CPT1C-mediated AMPAR effect. **(A)** Western blot showing that mutated CPT1C(H470A) interacts with GluA1. Anti-GFP antibodies could pull-down GluA1 when expressed together with CPT1C orCPT1C(H470A), (middle lanes, upper panel). GluA1 recognizing antibodies pull down both CPT1C and CPT1C(H470A), (right lanes, lower panel). This experiment was replicated three times. **(B)** Whole-cell GluA1-mediated currents between –80 mV and +80 mV in three example cells with similar membrane capacitance transfected with GluA1 alone (GluA1+GFP; left trace), together with CPT1C (middle trace) or CPT1C(H470A), (right trace). **(C)** Representative confocal images of tsA201 cells co-transfected with GluA1+GFP (left panel), GluA1+CPT1C (middle panel) or GluA1+CPT1C(H470A), (right panel). Surface and intracellular GluA1 were labeled with anti-GluA1-NT and Alexaflour 555 (red signal) or Alexaflour 647 (blue signal) as described in “Materials and Methods” section. Scale bars: 50 μm. **(D)** Average of normalized currents at –80 mV for the three conditions shown in **(B)**. CPT1C increased GluA1-mediated currents (**p < 0.01; Mann-Whitney-U-test) while removal of catalytic His 470 in CPT1C avoided the enhancement of the currents (^#^*p* < 0.05; Mann-Whitney-U-test). Numbers in bars denote the number of recordings. **(E)** Quantification of the GluA1 surface to intracellular ratio normalized to GluA1 and expressed as a percentage. The increment in surface expression due to CPT1C (**p < 0.01; Mann-Whitney-U-test) was abolished when catalytic His 470 was neutralized (^###^*p* < 0.001; Mann-Whitney-U-test). Numbers in bars denote the number of analyzed cells from three different IF experiments.

Given that the mutation of the catalytic triad abolishes the CPT1C effect on GluA1, we next decided to focus on one of these residues to test whether it was crucial for its activity. Given the importance of His 473 in CPT1s activity (Morillas et al., 2001), we used site-directed mutagenesis to create a mutant version of CPT1C where this important histidine was changed to alanine (H470A in CPT1C) to allow us to check if this residue alone is crucial for the CPT1C effect on GluA1. As previously done for CPT1C(CatTriad), we performed coimmunoprecipitation assays to evaluate the correct interaction between GluA1 and CPT1C(H470A). As expected from the results obtained in Figures 2D, 4A shows that both proteins still interact regardless of the mutation introduced to CPT1C. We then did electrophysiological and IF experiments to test the ability of CPT1C(H470A) to modulate GluA1 currents. Patch-clamp recordings such as the ones presented in Figure 4B showed an increase in AMPAR whole-cell current densities at –80 mV with CPT1C (82.39 ± 15.83 pA/pF for GluA1 alone vs. 149.7 ± 19.98 pA/pF GluA1+CPT1C; *p* = 0.0051; Mann–Whitney U-test; *n* = 25 and 19, respectively; Figures 4B,D). As hypothesized, CPT1C(H470A) did not increase GluA1 mediated currents (82.39 ± 15.83 pA/pF for GluA1 vs. 78.98 ± 18.26 pA/pF for GluA1+CPT1C(H470A); *p* = 0.6209; Mann–Whitney U-test; *n* = 25 and 20, respectively; Figure 4D), indicating that the His 470 residue plays a crucial role in GluA1 subunit modulation. In the same line, IF analysis (Figure 4C) showed that the increase in GluA1 surface/intracellular ratio observed with CPT1C (100.0 ± 5.3% for GluA1 vs. 123.5 ± 5.25% for GluA1+CPT1C; *p* = 0.0033; Mann–Whitney U-test; *n* = 104 and 94, respectively from three independent IF; Figure 4E) was not present with CPT1C(H470A; 123.5 ± 5.25% for GluA1+CPT1C vs. 102.2 ± 4.04% for GluA1+CPT1C(H470A); *p* = 0.0022; Mann–Whitney U-test; *n* = 104 and 147, respectively from three independent IF; Figure 4E) indicating that His 470 is crucial for CPT1C-mediated GluA1 surface trafficking enhancement.

### Molecular Model of CPT1C Complexed to Carnitine, CoA and Palmitate

**Figure 5:**
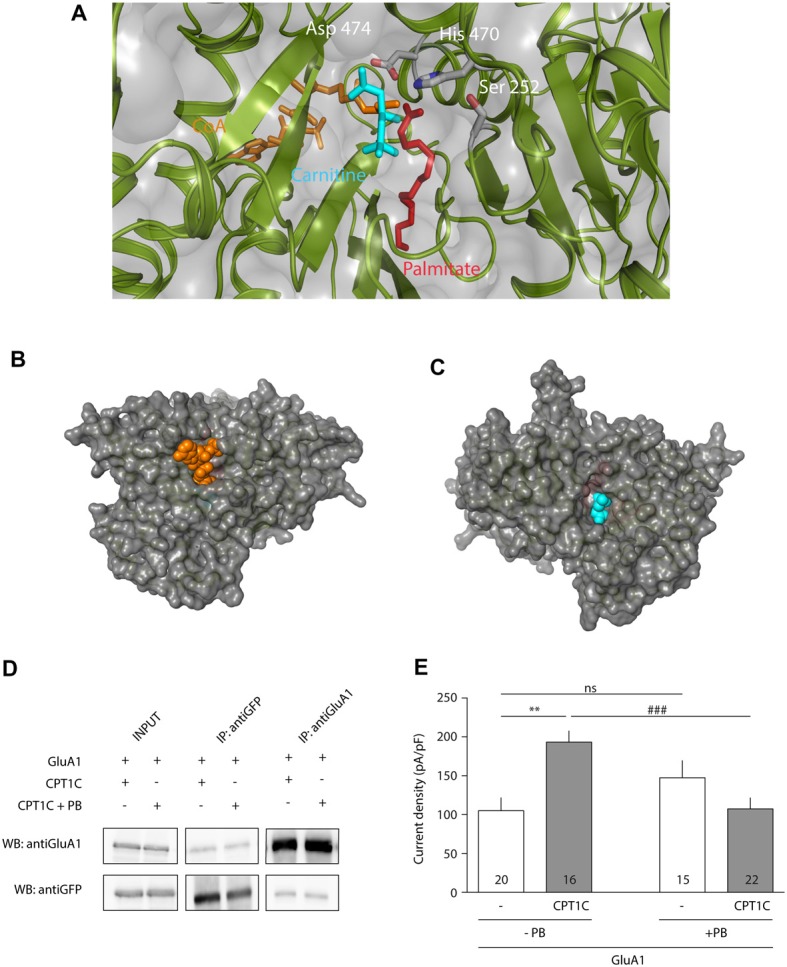
CPT1C displays depalmitoylation features. **(A)** Molecular model of CPT1C complexed to carnitine, CoA and palmitate. Ser 252, His 470 and Asp 474 are pointing toward the binding pocket and are proposed to be the catalytic residues in CPT1C depalmitoylating activity. **(B)** CoA (in orange) is observed in CPT1C molecular model, suggesting that the substrate (small region of GluA1) could access to the binding pocket through this cavity. **(C)** A cavity next to carnitine (in blue) is also observed, suggesting that palmitoylcarnitine can exit the binding pocket through this cavity. **(D)** Coimmunoprecipitation showing that Palmostatin B (PB)—an inhibitor of palmitoyl thioesterase (PTE) activity—treatment does not affect the interaction of CPT1C with GluA1. **(E)** Average of normalized current density at −80 mV for GluA1 alone (+GFP) or together with CPT1C with and without treatment with PB. CPT1C increased GluA1 current density in the absence of PB (**p < 0.01; Mann-Whitney-U-test). Current density was decreased to control levels (GluA1 alone) when cells co-expressed GluA1 and CPT1C but treated with PB (^###^*p* < 0.001; Mann-Whitney-U-test). There was no statistical difference in GluA1 current density when expressed alone, with or without PB treatment (^ns^*p* > 0.05; Mann-Whitney-U-test). Numbers in bars represent the number of recorded cells.

Once the importance of the triad—and His 470—in CPT1C effect on GluA1 had been assessed, we further tested the hypothesis that CPT1C is a depalmitoylating enzyme of AMPARs. Figure 5A shows the structural molecular model of human CPT1C complexed to carnitine, CoA and palmitate, where Ser 252, His 470 and Asp 474 point towards carnitine and palmitate molecules, reinforcing the hypothesis that CPT1C could perform a putative thioesterase activity. In addition, the molecular model shows that CoA and carnitine are highly accessible in the CPT1C structure (see Figures 5B,C respectively), suggesting that CPT1C would be able to bind a region of GluA1 instead of CoA.

### CPT1C Effect on AMPARs Is Abolished by Treatment With the Protein Thioesterase Inhibitor Palmostatin B

Since PB is a potent inhibitor of APT1 activity (Dekker et al., 2010), and considering the high structural homology between the APT1 and CPT1C catalytic triads, we decided to use this compound as a tool to investigate the role of palmitoylation in AMPAR modulation by CPT1C protein. We first did a coimmunoprecipitation assay to check if this compound could affect the AMPAR-CPT1C interaction. Cells were transiently transfected with GluA1 and CPT1C-GFP and 24 h later were treated during a time period of 24 h with either 50 μM PB or 0.1% DMSO (vehicle used to resuspend a 50 mM stock solution of PB). Afterwards, the compound was removed and the membranes of the transfected cells were immunoprecipitated with anti-GFP or with anti-GluA1. Figure 5D shows that treatment with PB does not alter GluA1 and CPT1C interaction. After confirming the interaction of GluA1 and CPT1C with Co-IP experiments, electrophysiological recordings were performed to evaluate the possible effect of PB on GluA1 mediated currents when co-expressed with CPT1C (Figure 5E). As control, in non PB treated cells, CPT1C increased GluA1 current density (105.1 ± 16.94 pA/pF for GluA1 vs. 193.2 ± 14.58 pA/pF for GluA1+CPT1C; *p* = 0.0011; Mann–Whitney U-test; *n* = 20 and 16 respectively). No statistical difference was found in GluA1 current density when expressed alone compared with the same condition but treated with PB (105.1 ± 16.94pA/pF vs. 147.4 ± 22.28 pA/pF; *p* = 0.1666; Mann–Whitney U-test; *n* = 20 and 15 respectively). Interestingly, PB treatment abolished the increase in current density due to CPT1C co-expression (193.2 ± 14.58 pA/pF for non-treated GluA1+CPT1C transfected cells vs. 107.4 ± 14.36 pA/pF for PB treated GluA1+CPT1C cells; *p* = 0.0007; Mann–Whitney U-test; *n* = 16 and 22 respectively). This experiment indicates that PB suppresses the GluA1-enhancing properties of CPT1C most probably by inhibiting its PTE activity.

### H470A Mutation Impairs CPT1C Activity in Hippocampal Neurons

We next decided to explore the role played by histidine 470 of CPT1C in neurons. Thus, we prepared primary neuronal cultures from CPT1C KO mice in which we overexpressed either WT CPT1C or CPT1C(H470A) and studied both GluA1 levels by IF and AMPAR-mediated currents with electrophysiology.

**Figure 6:**
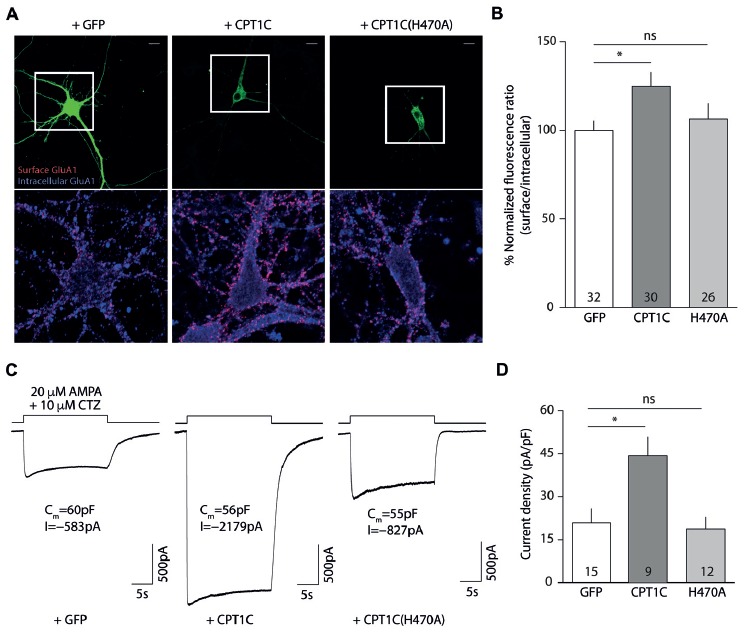
CPT1C but not CPT1C(H470A) increases functional AMPAR surface expression in CPT1C KO pyramidal neurons. **(A)** Representative confocal IF images of 11 DIV hippocampal pyramidal neurons in culture from CPT1C KO mice transfected with GFP (left images), CPT1C (middle images) orCPT1C(H470A), (right images). Upper panels display the analyzed transfected neuron. Surface GluA1 (Alexafluor 555; red signal) and intracellular GluA1 (Alexafluor 647; blue signal) is shown in the lower panels for the three different conditions. Scale bars: 10 μm. **(B)** Quantification of endogenous somatic GluA1 surface to intracellular ratio from CPT1C KO pyramidal cells in culture. GluA1 ratio was increased in neurons transfected with CPT1C compared with GFP (*p = 0.0132; Mann-Whitney U-test) while CPT1C(H470A) was not able to significantly increase GluA1 ratio (^ns^*p* = 0.8551; Mann-Whitney U-test). Numbers in bars indicate the number of neurons analyzed. **(C)** Endogenous AMPAR somatic currents evoked by rapid application of 20 μM AMPA +10 μM cyclothiazide during 20 s from CPT1C KO hippocampal pyramidal neurons transfected with GFP (left trace), CPT1C (middle trace) or CPT1C(H470A), (right trace). Three neurons with similar cell capacitance are shown. **(D)** Quantification of peak responses (shown as current density) from CPT1C KO pyramidal neurons in the conditions mentioned in **(C)**. Currents were clearly increased in neurons expressing CPT1C but not in cells expressing CPT1C(H470A). Numbers inside bars denote the number of recorded cells.

Figures 6A,B shows how CPT1C expression in KO neurons rescued GluA1 somatic levels compared with GFP expressing neurons measured by IF (100.00 ± 5.36% vs. 124.9 ± 7.67% for GFP and CPT1C respectively; *p* = 0.0132, Mann-Whitney U-test; *n* = 32 and 30 neurons). The increase in GluA1 surface expression was not significantly different from GFP expressing neurons when the mutated CPT1C(H470A) was used instead of WT CPT1C (100.00 ± 5.36% vs. 106.5 ± 8.55% for GFP and CPT1C(H470A) respectively; *p* = 0.8551, test; *n* = 32 and 26 neurons).

These results point to a role for histidine 470 in CPT1C in mediating the increase in AMPARs to the cell surface. However, in order to functionally assess the impact of the mutation, we recorded whole-cell somatic AMPAR currents. We rapidly applied AMPA plus cyclothiazide to the soma of hippocampal pyramidal neurons and recorded the evoked currents (Figure 6C). CPT1C enhanced the AMPAR-mediated responses (20.86 ± 4.93 pA/pF vs. 44.28 ± 6.64 pA/pF for GFP and CPT1C respectively; *p* = 0.0101 Mann-Whitney U-test; *n* = 15 and 9) in contrast to CPT1C(H470A) where no difference from the control cells was observed, confirming the requirement of the CPT1C catalytic histidine for the increase of the currents (20.86 ± 4.93 pA/pF vs. 18.77 ± 4.15 pA/pF for GFP and CPT1C(H470) respectively; *p* = 0.9641 Mann-Whitney U-test; *n* = 15 and 12) as shown in Figure 6D.

### CPT1C Possess Protein Thioesterase Activity Which Is Dependent on Residue H470

So far, the results obtained with CPT1C(H470A) seem to indicate that this protein exerts its effects on GluA1-containing AMPARs by a depalmitoylating mechanism. Consistent with this, CPT1C enhancement of surface AMPARs depends on the palmitoylable residue of GluA1, C585 (Gratacòs-Batlle et al., 2015). However, in the past, the putative depalmitoylating activity of CPT1C was assessed by means of an ABE assay without clear conclusions (Gratacòs-Batlle et al., 2015). We addressed the CPT1C depalmitoylating activity by means of an ABE assay in a HEK293-AD line constitutively expressing GluA1 (HEK293-GluA1) and overexpressing the palmitoyl acetyl transferase—DHHC3/GODZ—that palmitoylates the C585 residue of GluA1 (Hayashi et al., 2005). We reasoned that if basal GluA1 palmitoylation levels on C585 were higher the putative depalmitoylation effect of CPT1C would be more easily observed.

**Figure 7:**
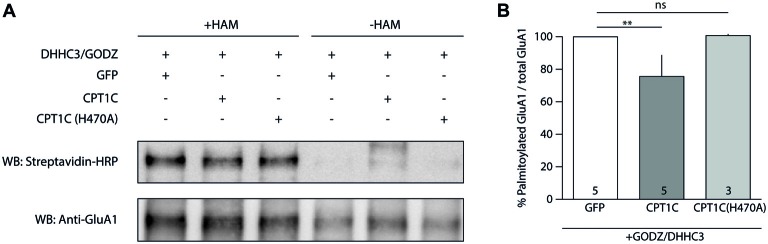
CPT1C acts as depalmitoylating enzyme of GluA1. **(A)** Palmitoylation levels detected with Acyl-Biotin Exchange (ABE) assay of GluA1 alone (GFP), or together with CPT1C-GFP or CPT1C(H470A) in HEK293AD-GluA1 expressing cells transfected with DHHC3/GODZ palmitoylating enzyme. The biotinylated GluA1 immunoprecipitates subsequent to the ABE assay for all conditions were subjected to SDS-PAGE. Palmitoylation of GluA1 subunit is detected only in plus-hydroxylamine (+HAM) samples (three lanes from the left). −HAM samples control non-specific incorporation of biotin (three lanes from the right). GluA1 palmitoylation levels (right top) were detected by Western blotting with streptavidin-HRP (palmitoylation). The total amount of immunoprecipitated GluA1 was detected by Western blotting with anti-GluA1-NT antibody (anti-GluA1, bottom) after stripping the membranes. **(B)** Quantification of palmitoylation levels for GluA1 alone (GFP), together with CPT1C or CPT1C(H470A) in HEK293AD cells constitutively expressing GluA1. Ratio of palmitoylated GluA1 to total GluA1 is shown as mean and S.E.M. (**p < 0.01 and ^ns^*p* > 0.05; Mann-Whitney U test; *n* = 5, 3 and 3, respectively).

Hence, we transfected HEK293-GluA1 cells with either GODZ+GFP or GODZ+CPT1C and we performed the ABE assay (Figure 7A). As hypothesized, we observed a decrease in palmitoylation levels of GluA1 subunit when CPT1C was present (100% for GODZ+GFP vs. 75.59 ± 13.04% for GODZ+CPT1C; *p* = 0.0079; Mann-Whitney U-test; *n* = 5; Figure 7B). This directly demonstrates that CPT1C is able to decrease GluA1 palmitoylation. Importantly, cells expressing the mutant H470A form of CPT1C had GluA1 palmitoylation levels similar to those of GFP transfected cells indicating that GluA1-dependent depalmitoylation by CPT1C relies on its catalytic residue histidine 470 (100% for GODZ+GFP vs. 100.70 ± 0.78% for GODZ+CPT1C(H470A); *p* = 0.4643; Mann-Whitney U-test; *n* = 5 and 3, respectively; Figure 7B). These experiments demonstrate that CPT1C is able to remove palmitate groups from GluA1 and that this catalytic activity depends on histidine 470.

### CPT1C Effect on GluA1 Is Isoform Specific and Restricted to Endoplasmic Reticulum

**Figure 8:**
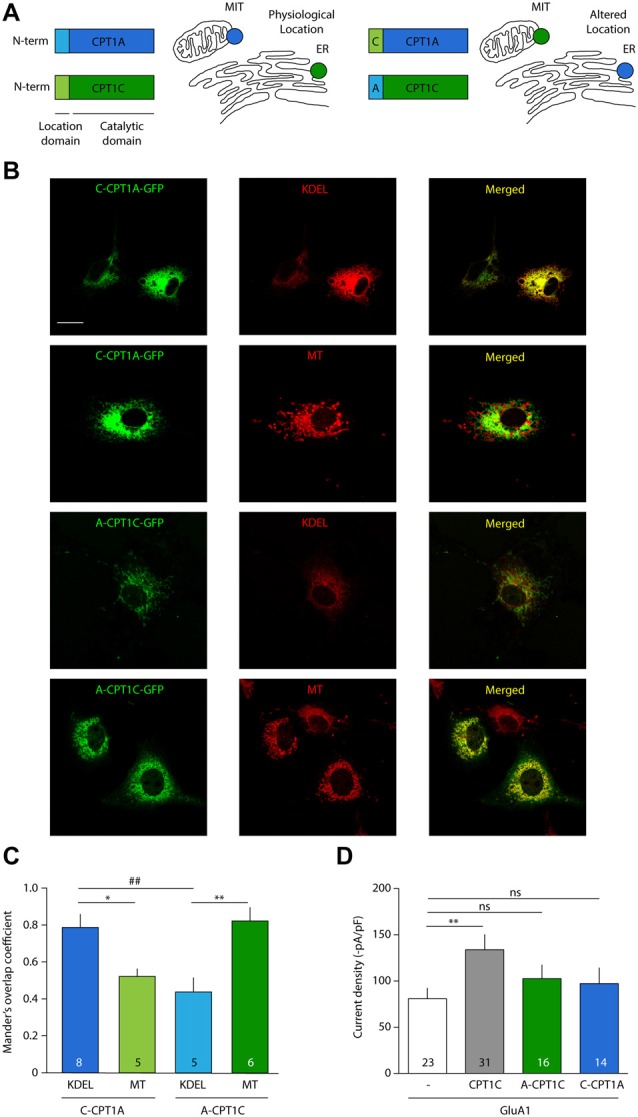
CPT1C effect on AMPAR currents is isoform specific and depends on ER location. **(A)** N-terminal domain of CPT1s determines its intracellular location. Chimeric proteins *C*-CPT1A (CPT1A with the location domain of CPT1C) and *A*-CPT1C (CPT1C with the location domain of CPT1A) are miss-localized to endoplasmic reticulum (ER) and mitochondria (MIT/MT), respectively. **(B)** Confocal images showing CPT1s-GFP signal in green (left columns), KDEL or mitotracker signal in red (middle column) and merged images (right columns) for the two chimeric CPT1s proteins. *C*-CPT1A clearly co-localizes with ER while *A*-CPT1C is not co-localizing with KDEL ER marker. **(C)** Representation of co-localization values quantified by Manders’ overlap Coefficient (MOC) expressed as mean ± SEM. Numbers in bars denote number of analyzed cells (*p < 0.05; **, ^##^*p* < 0.01; Mann-Whitney-U-test). **(D)** Average of normalized current density at –80 mV for GluA1 alone or together with CPT1C or chimeric proteins. No significant differences were found between GluA1 current density (–pA/pF) in cells co-expressing *A*-CPT1C or *C*-CPT1A (^ns^*p* > 0.05; Mann-Whitney-U-test).

It has been shown in the past that CPT1A does not share the ability of CPT1C to modulate AMPAR surface expression (Gratacòs-Batlle et al., 2015). We next decided to explore whether the effect of CPT1C on AMPARs is due to its specific ER localization and whether CPT1A could exert a similar effect on AMPAR mediated currents when targeted to ER. For this purpose we took advantage of the fact that the N-terminus of CPT1s is responsible for targeting CPT1C and CPT1A to specific intracellular locations (Sierra et al., 2008). Concretely, while the CPT1C N-terminal domain determines its ER targeting, the N-terminal domain of CPT1A contains a mitochondrial import signal (Cohen et al., 2001), which is responsible for its mitochondrial location (Figure 8A; left). Two chimeric proteins were used, where the N-terminal region of CPT1C was cloned into the CPT1A-GFP plasmid (named hereafter: *C*-CPT1A) and the N-terminal region of CPT1A replaced the equivalent region in the CPT1C-GFP plasmid (named hereafter: *A*-CPT1C), thus switching their intracellular locations (Figure 8A; right).

First, to confirm CPT1A and CPT1C mislocalization, these constructs were transfected in COS-7 cells. Co-localization imaging experiments were performed with these constructs and MitoTracker (a potential-sensitive dye that accumulates in mitochondria) or KDEL-DsRed (ER marker). As can be observed in the confocal photo-micrographs in Figure 8B, CPT1A with the localization motif of CPT1C (*C*-CPT1A) shows a clear ER pattern (six upper panels), meanwhile CPT1C with the localization domain of CPT1A (*A*-CPT1C) localizes at the mitochondria (six lower panels). Quantification of these localizations was performed by using MOC as shown in Figure 8C. Hence, we confirmed that the subcellular locations of CPT1C and CPT1A could be changed by switching their location motifs. Next, in order to assess the functional consequences of CPT1A and CPT1C on GluA1 when they are in a different subcellular location from their usual one, electrophysiological whole-cell recordings were carried out using tsA201 cells expressing GluA1 alone, in presence of CPT1C or with chimeric *C-CPT1A and* *A*-CPT1C. As seen before, Figure 8D shows that GluA1 current density was lower in cells expressing GluA1 alone when compared with cells expressing GluA1 together with CPT1C (80.83 ± 11.75 pA/pF vs. 133.9 ± 16.29 pA/pF; *p* = 0.0062; Mann–Whitney U-test; *n* = 23 and 31, respectively). GluA1 current density in cells expressing GluA1 alone was similar to the current density of cells co-expressing miss-localized CPT1C *(A*-CPT1C; 80.83 ± 11.75 pA/pF vs. 102.7 ± 16.29 pA/pF; *p* = 0.1571; Mann–Whitney U-test; *n* = 23 and 16, respectively) indicating that the effect of CPT1C depends on its ER location. Interestingly, no differences were found when comparing cells expressing GluA1 alone or with GluA1 co-expressed with CPT1A at ER location (*C*-CPT1A; 80.83 ± 11.75 pA/pF vs. 97.24 ± 16.97 pA/pF; *p* = 0.1571; Mann–Whitney U-test; *n* = 23 and 14, respectively) indicating the inability of CPT1A to act on AMPARs.

Palmitoylation of AMPARs occurs at the Golgi Apparatus (GA) by DHHC3/GODZ on Cysteine 585 (Hayashi et al., 2005). To date no DHHCs capable of palmitoylating GluA subunits have been described to be present at the ER and hence palmitoylate AMPARs at early stages. This raises the question of how CPT1C—an ER resident protein—can depalmitoylate a protein that is normally being palmitoylated at the GA. One possibility is that both processes occur sequentially in the transition between ER and GA, which is performed via COPII vesicles (Watson and Stephens, 2005). In fact, anterograde transport of GluA1 from ER to Golgi, which is mediated by COPII vesicles, is important for GluA1 palmitoylation in neurons (Yang et al., 2009). Thus, we next wanted to investigate whether CPT1C was present in these vesicles apart from the ER, which would allow depalmitoylation of GluA1 at COPII vesicles. To test that hypothesis, we performed co-localization experiments of CPT1C-GFP with Sec31A (COPII marker). MOC showed values of 0.62 ± 0.03 (*n* = 15; Supplementary Figure S3), which were not conclusive. Thus, to determine if this MOC value was indicating the presence of CPT1C in COPII vesicles, we performed co-localization experiments between Sec31A (COPII marker) and Sec61B-GFP (ER marker; Johnson and van Waes, 1999). We obtained a MOC value of 0.61 ± 0.003 (*n* = 9; Supplementary Figure S2), which was not significantly different from the MOC value between CPT1C and Sec31A (*p* = 0.7622) suggesting that this value is due to the close proximity of the ER to COPII vesicles and not due to the specific presence of CPT1C in COPII.

In summary, this data indicates that the depalmitoylating effect of CPT1C on AMPARs is clearly CPT1C specific and possibly happens at ER level.

## Discussion

For many years, it has been known that AMPARs are key elements in synaptic function. However, during the last decade, the AMPAR field has been transfigured by the gradual discovery of transmembrane proteins interacting with AMPARs that regulate and determine their function in the brain. In 2012, proteomic studies (Schwenk et al., 2012; Shanks et al., 2012) defined several new proteins that were able to interact with AMPAR subunits. We previously described a novel role for one of these proteins (CPT1C) in GluA1-containing AMPAR function (Gratacòs-Batlle et al., 2015) whereby CPT1C increases the surface expression of this ionotropic glutamate receptor subtype. Since prior results indicate that CPT1C fails to promote GluA2 homomeric AMPAR trafficking (Gratacòs-Batlle et al., 2015), in this work we have focused on GluA1 subunit of AMPARs due to the apparent subunit specificity of CPT1C effect. However, future experiments might try to resolve whether other AMPAR subunits (GluA3 and GluA4) can be targeted by CPT1C and which could be the determinants of the differences found between GluA1 and GluA2. Our results reinforce the evidence of CPT1C importance in AMPAR regulation by showing that GluA1 clearly coimmunoprecipitates with CPT1C in brain extracts from cortex, cerebellum or hippocampus and modulates somatic AMPAR content in pyramidal neurons. The interaction presented here confirms previous reports of GluA1-CPT1C interaction from mouse/rat whole brain extracts in proteomic studies (Schwenk et al., 2012, 2014) and from mouse hippocampal neuronal cultures (Fadó et al., 2015). Interestingly, our coimmunoprecipitation data from brain extracts in Figure 1 show lower amounts of GluA1 protein in all tested brain areas in CPT1C KO animals compared with WT animals. In this regard, previous studies have attributed a role for CPT1C in stabilizing and controlling GluA1 synthesis in cultured HPNs (Fadó et al., 2015). Our results reinforce this hypothesis and suggest a similar decrease in expression for GluA1 in cerebellar and cortical neurons of CPT1C deficient animals (Figure 1A, left panel). CPT1C function in the central nervous system (CNS) has been demonstrated to be important in hypothalamic control of food intake (Wolfgang et al., 2006) and in hippocampal function as can be inferred from the learning deficits in CPT1C KO animals (Carrasco et al., 2012). Specifically, CPT1C-lacking mice display poor performance in the Morris water maze (Carrasco et al., 2012)—a task that clearly explores hippocampal function but also depends on cortical areas (D’Hooge and De Deyn, 2001). Moreover, CPT1C deficient mice have motor coordination impairment (Carrasco et al., 2013), which is indicative of cerebellar malfunctioning. Our data showing a widespread GluA1-CPT1C interaction in the CNS and a deficit of somatic AMPARs in hippocampal and cortical neurons from CPT1C KO animals reinforces the idea that CPT1C plays an important role in modulating AMPARs in these brain regions.

However, the main scope of this work has been focused on resolving the molecular mechanisms of CPT1C modulation of AMPARs. Given that CPT1C binds palmitoyl-CoA and is able to form palmitoylcarnitine (Sierra et al., 2008), we considered whether CPT1C could depalmitoylate GluA1, which would explain the increase in AMPAR surface expression. Palmitoylation/depalmitoylation of C585 and C811 in GluA1 determine trafficking properties and surface stability of AMPARs (Hayashi et al., 2005; Yang et al., 2009; Lin et al., 2009). Indeed, the palmitoylable cysteine 585 of the GluA1 subunit has been proved to be crucial for the CPT1C enhancement of AMPAR trafficking although no changes in the palmitoylation state of GluA1 could be observed (Gratacòs-Batlle et al., 2015). In order to gain insight into the functional role of CPT1C, we constructed a structural model of *human* CPT1C as a tool to elucidate the molecular mechanism of a putative depalmitoylating activity. The structural similarity of the CPT1C molecular model and protein thioesterases permitted the identification of Ser 252, His 470 and Asp 474 as the catalytic triad responsible for depalmitoylating activity in CPT1C. We have also constructed a structural molecular model of CPT1C complexed to carnitine, CoA and palmitate, where the side-chains of the residues from the catalytic triad point towards these molecules (see Figure 5A). Based on this structural model, the palmitoylated substrate (a small region of GluA1) would access the binding pocket through the CoA cavity (Figure 5B) exposing the palmitate moiety to the catalytic triad.

AMPARs at ER are positioned in such a way that their palmitoylation sites are facing the cytoplasmic side (Gan et al., 2015). The catalytic domains of CPT1A and B face the cytoplasm (Fraser et al., 1997) and this is also the case for CPT1C (Supplementary Figure S4). This topology of CPT1C at the ER would allow contact between the CPT1C catalytic domain and the palmitoylable cysteine residues of the AMPARs. The residues from the catalytic triad of CPT1C domain would perform the thioesterase activity, thus transferring the palmitoyl moiety from the substrate (GluA1) to the carnitine molecule. The resulting palmitoylcarnitine molecule would be able to exit through another cavity opposite to the GluA1 binding pocket (corresponding to the carnitine cavity, Figure 5C). Experiments performed with two catalytic mutant versions of CPT1C—CPT1C(H470A) and CPT1C(CatTriad)—validate our model. First, in these CPT1C versions, the increase in AMPAR surface expression mediated by CPT1C was abolished indicating that the effect of CPT1C on GluA1 subunits was most probably mediated by the depalmitoylating activity of CPT1C. Second, PB, a compound known to specifically inhibit APT1 thioesterase activity (Dekker et al., 2010), was able to preclude the increment in AMPAR-mediated responses, strongly suggesting that CPT1C acts as a depalmitoylating enzyme, and third, this work directly demonstrates the depalmitoylating activity of CPT1C, which probably accounts for the effect on AMPAR traffic enhancement. Previous results hinted at the possible depalmitoylating role of CPT1C since its effect on AMPARs was abolished in the non-palmitoylable version of GluA1 subunit, GluA1(C585S; Gratacòs-Batlle et al., 2015). However, this previous work failed to demonstrate a depalmitoylating activity of CPT1C. Similarly, other works did not found a putative CPT1 catalytic activity for CPT1C equivalent to CPT1A and CPT1B (Sierra et al., 2008). Here we have tried to improve the ABE assay sensitivity by decreasing the variability in basal palmitoylation levels of GluA1 subunits, which might account for the variability found in earlier work (Gratacòs-Batlle et al., 2015). We have used DHHC3/GODZ—a specific palmitoyl acetyl transferase that palmitoylates GluA1 at cysteine residue 585 (Hayashi et al., 2005)—to increase the palmitoylation levels of GluA1 in order to reliably detect depalmitoylating activity of CPT1C. Under these conditions, we were able to observe differences in the palmitoylation levels of GluA1. The fact that CPT1C(H470A) did not alter the palmitoylation levels of GluA1 compared with control conditions supports the depalmitoylating activity of CPT1C and points towards the importance of this catalytic histidine in the depalmitoylation process. This is not surprising since mutations affecting the catalytic histidine of LYPLA1, a thioesterase that depalmitoylates the BKCa channels, impair the depalmitoylation activity of this enzyme (Tian et al., 2012).

The rescue experiments performed in this work reinforce the importance of CPT1C—and its catalytic activity—in neuronal physiology. In CPT1C deficient hippocampal pyramidal neurons, the re-expression of CPT1C increased the surface/intracellular ratio of GluA1 while CPT1C(H470A) did not. Furthermore, CPT1C(H470) failed to upregulate AMPAR-mediated currents in this same neuronal type when CPT1C clearly increased the somatic currents.

Thus, collectively our experiments prove the involvement of a depalmitoylation process in the CPT1C-mediated increase of surface AMPARs and also show that CPT1C acts on AMPAR through an enzymatic activity (dependent on a catalytic histidine) and not due to a mere structural interaction. This function of CPT1C is maintained in neurons since neutralization of the catalytic residue H470 abolishes the ability of CPT1C to exert its effect on native AMPARs from hippocampal pyramidal cells.

Previous studies have shown that GluA1 modulation is specific to the CPT1C isoform. The possibility that CPT1A could mediate the same effect as CPT1C on AMPAR function was previously explored (Gratacòs-Batlle et al., 2015) due to the high degree of homology between both isoforms (Price et al., 2002) and concluded that CPT1A was incapable of modulating AMPAR trafficking. Nevertheless, the subcellular locations of CPT1A and GluA1 are totally different and perhaps this is the basis for the lack of effect of CPT1A on GluA1 trafficking. Here we have circumvented that issue by targeting CPT1A to the ER, where it can be in direct contact with GluA1. Our results show that when CPT1A localizes at the ER together with GluA1 it does not modulate AMPAR-induced currents as CPT1C does, confirming the specificity of this member of the CPT1 family and supporting the hypothesis of a different enzymatic activity for CPT1C. On the other hand, the ER location of CPT1C seems to be an essential requirement for modulating AMPAR surface expression as mislocalization of CPT1C abolishes its effect on AMPAR currents. Co-localization studies have shown that GluA1-CPT1C interaction only occurs at ER level and not at other cell locations as GA or cell surface (Gratacòs-Batlle et al., 2015). Moreover, it has been recently shown that CPT1C binds exclusively to a pool of AMPARs that are devoid of auxiliary subunits as CNIHs or TARPs. This population of AMPARs is only found at the ER and not at other cell locations as GA or plasma membrane (Brechet et al., 2017), reinforcing the idea presented here that the CPT1C mediates its effects exclusively at the ER. This conclusion implies that a palmitoyl acyl transferase (putatively a member of DHHC family) should be present at ER, and to date no DHHC proteins capable of palmitoylating GluA1 have been found at ER. Another possibility is that palmitoylated AMPARs undergo retrograde traffic form the GA to the ER via COPI vesicles (Spang, 2013; Fleck, 2006). Indeed, palmitoylation by DHHC3 of GluA2 subunits on residue C610 at TM2 (the equivalent residue to C585 in GluA1) accumulates AMPARs at the GA (Hayashi et al., 2005), which might favor their retrograde transport to the ER and thus make them a target for CPT1C depalmitoylation. The significance of palmitoylation at the GA and such putative AMPAR retrograde traffic is unknown, but it may form part of AMPAR quality control mechanisms (Penn et al., 2008; Coleman et al., 2010). Future investigation might resolve this caveat.

Together with CPT1C, a series of proteins forming part of the peripheral core of AMPAR complex have been described recently to affect AMPAR trafficking: porcupine protein has been shown to control AMPAR levels at the synapse (Erlenhardt et al., 2016); FRRS1L and CPT1C cooperatively act to favor AMPAR traffic to plasma membrane by priming the complex for TARPs and CNIHs association (Brechet et al., 2017); α/β-hydrolase domain-containing 6 (ABHD6) has a negative role on AMPAR trafficking (Wei et al., 2016). All these proteins are not present in TARPed AMPARs at the cell surface (Brechet et al., 2017), and their role seem to be restricted to intracellular compartments. Our work confirms these previous findings of CPT1C’s role on AMPARs trafficking (Gratacòs-Batlle et al., 2015; Brechet et al., 2017) and sheds light on the mechanism of the positive trafficking effect on AMPARs by suggesting that CPT1C catalytically depalmitoylates GluA1 in the ER prior to transport to the GA. Intriguingly, in contrast with the apparent necessity for CPT1C catalytic activity necessity, the hydrolase activity of a monoacylglycerol lipase, ABHD6, was not required for its effects on AMPARs (Wei et al., 2016).

Apart from their physiological role, numerous studies have implicated AMPARs in neurological conditions (Cull-Candy et al., 2006; Zarate and Manji, 2008; Chang et al., 2012) and the implication of CPT1C in alterations that affect neurons and glia has also been reported (Cirillo et al., 2014; Rinaldi et al., 2015). Besides, AMPAR physiology seems to be altered in normal ageing (Henley and Wilkinson, 2013), one of the most challenging aspects of neuroscience research. The decline in normal intellectual capabilities associated with ageing is related in part to a reduced synaptic plasticity arising from changes in postsynaptic membrane constituents, such as AMPAR number and function. Certainly, aberrant AMPAR trafficking and consequent abnormal changes in synapses are a core feature in age-dependent cognitive decline (Henley and Wilkinson, 2013). It has been previously described that synaptic transmission is diminished in CPT1C KO pyramidal HPNs (Fadó et al., 2015). In agreement with the already described role of CPT1C in regulating AMPAR surface expression, we have demonstrated that somatic AMPARs in HPNs are also diminished in CPT1C KO animals, which would account for the lower content of AMPARs at the synaptic level reported previously (Fadó et al., 2015). Interestingly there is a decline in CPT1C expression with age (Carrasco et al., 2013) and since AMPAR trafficking is favored by CPT1C (Gratacòs-Batlle et al., 2015) and the receptor levels are also stabilized by this protein (Fadó et al., 2015), this gradual decrease in expression of CPT1C might compromise the normal synaptic function. Thus, CPT1C presents itself as a part of the normal AMPAR cell biology and lack of this protein might be associated with aging-related problems although this idea has not been tested.

In summary, the work performed on this article sheds light on the regulation of AMPAR function by CPT1C, which modulates AMPAR trafficking by a putative depalmitoylating mechanism.

## Conclusion

Our results show for the first time that CPT1C possesses a depalmitoylating catalytic activity on GluA1 subunit of AMPARs. Based on *in silico* homology modeling of CPT1C structure, we have identified a catalytic triad also present in other PTE proteins. The histidine 470 from this catalytic triad of CPT1C seems to be crucial in this enzymatic activity. Mutation of His470 for an alanine abolishes both depalmitoylating activity on GluA1 and CPT1C-mediated AMPAR trafficking enhancement in cell lines and hippocampal neuronal cultures.

## Author Contributions

EG-B, MO and DS designed the work. EG-B, MO, NS-F, NY, FM-C, RF, MO and DS performed the research and analyzed the data. EG-B, MO, NC, XG, SA and DS interpreted the data. EG-B, MO and DS contributed to the manuscript writing.

## Conflict of Interest Statement

The authors declare that the research was conducted in the absence of any commercial or financial relationships that could be construed as a potential conflict of interest.

## Footnotes

^1^www.graphpad.com
