# Supplementary material for: Mechanisms of CPT1C-Dependent AMPAR Trafficking Enhancement
Source: Front Mol Neurosci. 2018 Aug 8;11:275. doi: 10.3389/fnmol.2018.00275 (PMC6092487; doi:10.3389/fnmol.2018.00275)
Supplement: Supplementary file 1 [file Data_Sheet_2.docx]

**SUPPLEMENTARY INFORMATION for:**

**Mechanisms of CPT1C-dependent AMPAR trafficking enhancement**

Esther Gratacòs-Batlle, Mireia Olivella, Nuria Sánchez-Fernández, Natalia Yefimenko, Federico Miguez-Cabello, Rut Fadó, Núria Casals, Xavier Gasull, Santiago Ambrosio and David Soto.

**Supplementary Figure 1: Negative control for brain tissue GluA1-CPT1C interaction.** Left panel: Input samples from cortex, cerebellum and hippocampus detected with antibodies against CPT1C (upper), GluA1 (middle) and actin (lower). Right panel: immunoprecipitated extracts with neutral IgGs or anti-GluA1 antibodies for each brain homogenate showing no signal in the neutral IgGs lanes and a clear band in anti-GluA1 precipitated samples. Signal was detected with anti-CPT1C antibody (upper) and anti-GluA1 antibody (lower).

Methods for supplementary Figure 1:

Hippocampi, cerebellums and frontal cortices were obtained from C57BL/6J wild-type mice of 2 months of age. When dissected, they were sonicated in cold Tris·HCl pH 7.4 (with Protease Inhibitor Cocktail (Roche) and PMSF) with a Broanson Sonifier 150 (5 watts). All subsequent steps were performed at 4 ºC. The homogenate was centrifuged at 16,000 xg for 30 min at 4 ºC. The pellet was resuspended by pipetting up and down in ice-cold lysis buffer (1 % IGEPAL, 50 mM Tris-HCl pH 7.5, 150 mM NaCl, 10 % Glycerol, Protease Inhibitor Cocktail (Roche) and PMSF) and dissolved during 30 min in an orbital agitator. Insoluble material was pelleted at 16,000xg for 30 min. The supernatant was quantified by the BCA method and 0.4-1 mg of protein were incubated overnight at 4ºC with 2 µg of anti-GluA1 antibody or 2 µg of neutral IgGs and 60 µl of Protein-A sepharose beads (Sigma) with orbital agitation. Antibody-protein complexes were washed with lysis buffer three times and eluted with 2x SB/5 mM DTT sample buffer, heated 10 min at 76 ºC and separated on SDS/PAGE. Before adding the antibodies, 10 % of total protein was removed as input samples and boiled at 75 °C for 10 min in 2x SB/5 mM DTT.

**Supplementary Figure 2: CPT1C deficiency translates into a lower surface expression of GluA1 in cortical neurons.**

(**A**) Immunofluorescence of 11DIV cortical neurons in culture from wild type (upper images) and CPT1C knockout (lower images) animals. Surface GluA1 (red signal) and intracellular GluA1 (blue signal) is shown.

(**B**) Quantification of endogenous somatic GluA1 surface to intracellular ratio from cortical cells in culture. GluA1 ratio was reduced in neurons from CPT1C knockout animals compared with wild type animals (***p=0.0006; Mann-Whitney-U-test). Numbers in bars indicate the number of neurons analyzed.

**Supplementary Figure 3: Colocalization experiments between CPT1C and COPII vesicles**

**(A)** Confocal image in COS-7 cells showing CPT1C-GFP signal in Green and Sec31A (COPII marker) signal in red, where only partial co-localization (yellow) is evident.

**(B)** Confocal image showing Sec61B (ER marker) signal in green and Sec31A (COPII marker) signal in red. As in (A), partial co-localization appears to be present.

**(C)** Mander’s Overlap Coefficient (MOC) co-localization values expressed as mean ± SEM. Numbers in bars denote number of analyzed cells Student-*t*-test.

**Supplementary Figure 4: CPT1C topology in the ER membrane**

**(A) Endogenous putative glycosylation sites of CPT1C.** Sites potentially exposed to the action of oligosaccharyltransferases are shown in green, and their respective amino acid positions are numbered on rat (Rn), mouse (Mm) or human (Hs) CPT1C sequences (**NST:** Asn-Ser-Thr, **NTT:** Asn-Thr-Thr and **NLS:** Asp-Leu-Ser). These sites were predicted using NetNGlyc 1.0 server (<http://www.cbs.dtu.dk/services/NetNGlyc>). The structure of the N-terminal domain (in red), the transmembrane domains (in brown) and region after transmembrane domains (in blue) are shown. The gap between the two transmembrane regions represents residues from 76 to 102 in Figure 2 that corresponds to the loop region.

**(B) Glycosilation sites at diferents regions of CPT1C.** Amino acid sequence of 150 first residues of rat CPT1C sequence: overview of the strategy used for introducing glycosylation sites at the N-terminal and loop region of CPT1C. **pIRES-Nterm** (i) incorporates a whole glycosylation site (**NIT**: Asn-Ile-Thr) introduced with the addition of 9 nucleotides into the CPT1C cDNA sequence and **pIRES-Loop** (ii) creates a glycosylation site (NIT) by replacing two nucleotides using site-directed mutagenesis. Predicted positions of both transmembrane regions are also shown.

**(C) Positive control of deglycosylation assay**. RNase B (a glycoprotein that contains a single glycosylation site at Asn34) was treated with or without PNGase F showing a mobility shift. Arrows point bands corresponding to glycosylated RNase B (“Glyc-RNAase B”, 16 kDa) and the deglycosylated form (“RNase B”, 13 kDa) in a 15% SDS-PAGE gel stained with Blue Coomassie.

**(D)** **Deglycosylation assay**. Immunoblotting with CPT1C antibody of microsomal fractions treated either with (+) or without (-) PNGase F and separated in 8% SDS-PAGE. Band corresponding to glycosylated form of CPT1C is indicated by an arrow and named as “Glyc-CPT1C”. Bands corresponding to non-glycosilated CPT1C are named “CPT1C”. Only microsomes expressing pIRES-Loop showed a mobility shift.

Methods for Supplementary Figure 4C-D (deglycosilation assay):

N-linked glycans are attached to the protein backbone via an amide bond to an asparagine residue in an Asn-Xaa-Ser/Thr motif, where X can be any amino acid, except proline. Variation in the degrees of saturation at available glycosylation sites results in heterogeneity in the mass and charge of glycoproteins. Although sites of potential N-glycosylation can be predicted from the consensus sequence Asn-Xaa- Ser/Thr, it cannot be assumed that a site will actually be glycosylated. Therefore the sites of glycan attachment must be identified and characterized by analytical procedures. Peptide-N-glycosidase F (PNGase F) (Sigma, ref. P7367) is one of the most widely used enzymes for the deglycosylation of glycoproteins. The enzyme releases asparagine-linked (N-linked) oligosaccharides from glycoproteins and glycopeptides. A tripeptide with the glycan-linked asparagine as the central residue is the minimum substrate for PNGase F.

The deglycosylation assay was performed diluting 160 µg of protein from microsomal fractions – obtained as described in (Sierra et al., 2008) – of HEK293T cells transfected with different constructs of pIRES2-EGFP (constructs that bear glycosylation sites in different regions of CPT1C protein: pIRES-Nterm, pIRES-Loop and pIRES-CPT1C, see Supplementary Figure 1A and 1B), in 20 mM ammonium bicarbonate, pH 8, to a final volume of 45 µl. For positive control, use 45 µl of RNase B (1.1 mg/ml) (Sigma, ref. R1153) (See Supplementary Figure 1C). 5 µl of denaturation solution (0.2% SDS, 100 mM 2-mercaptoethanol) were added to the extracts and samples were vortexed and heated to 100°C for 10min to denature proteins (in order to facilitate the access of the deglycosilating enzyme (PNGase F, Sigma, ref. P7367) to the N-glycans attached to the protein). After cooling the sample to room temperature 5 µl of 15% Triton X-100 and 10 µl of the prepared PNGase F enzyme solution (500U/ml) to the reaction mixture were added and incubated at 37 °C for 3 h adding 0.5 µl protease inhibitor mix every one hour. The reaction was stopped by heating the samples to 100 °C for 5min and 65 µl of 2x Protein sample buffer were added and prepared to analyze in SDS-PAGE electrophoresis using Western blot (Supplementary Figure 1D).
