# Supplementary figures and images for: Mechanisms of CPT1C-Dependent AMPAR Trafficking Enhancement
Source: Front Mol Neurosci. 2018 Aug 8;11:275. doi: 10.3389/fnmol.2018.00275 (PMC6092487; doi:10.3389/fnmol.2018.00275)

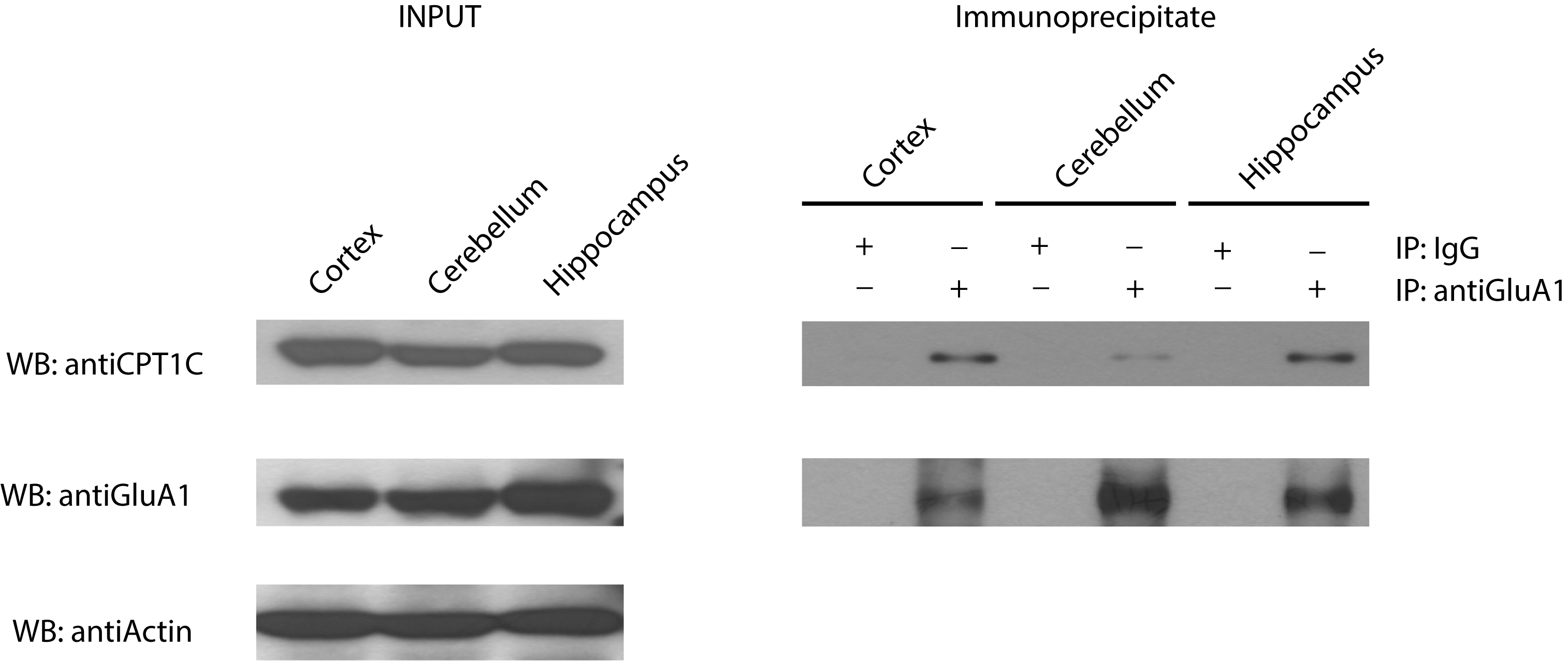

Supplement: Supplementary file 2 [file Image_1.TIF]

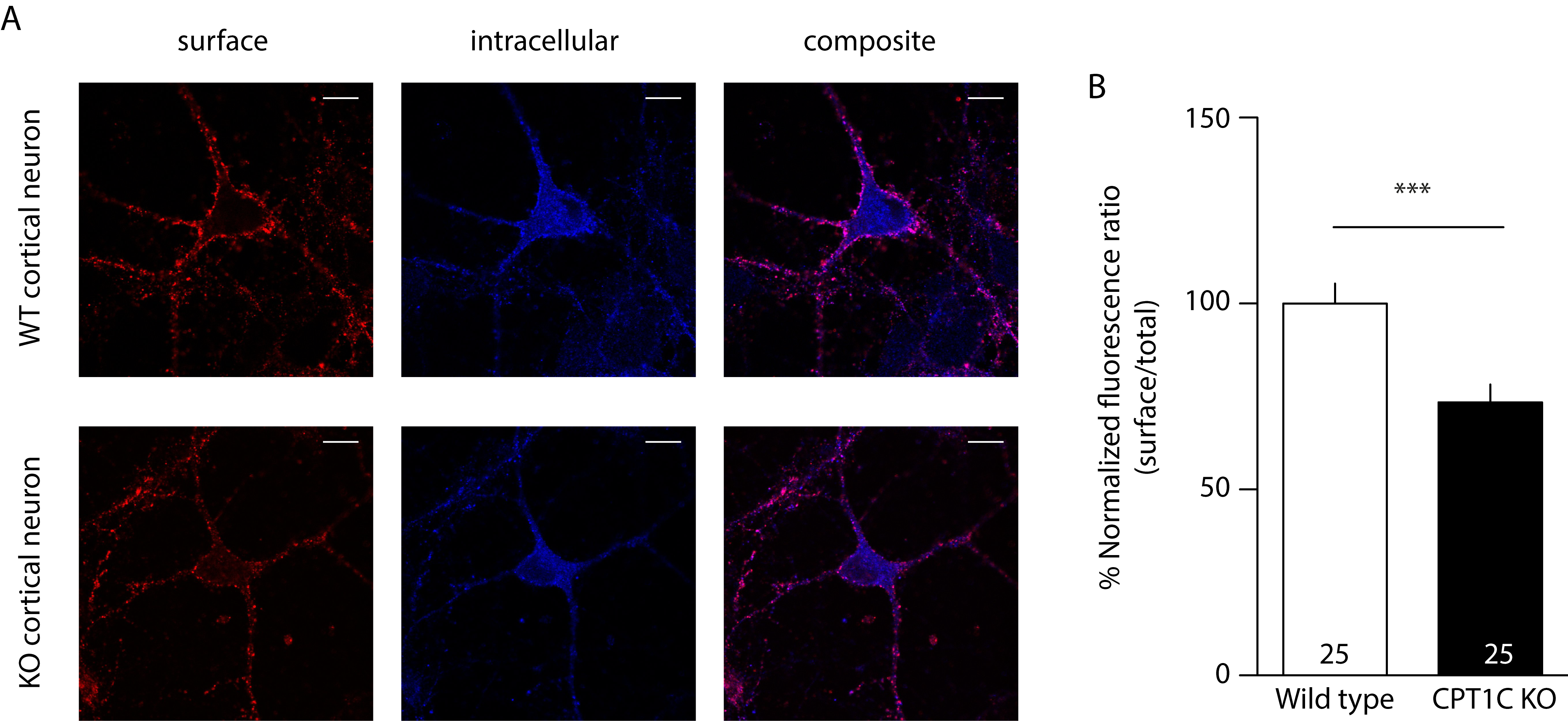

Supplement: Supplementary file 3 [file Image_2.TIF]

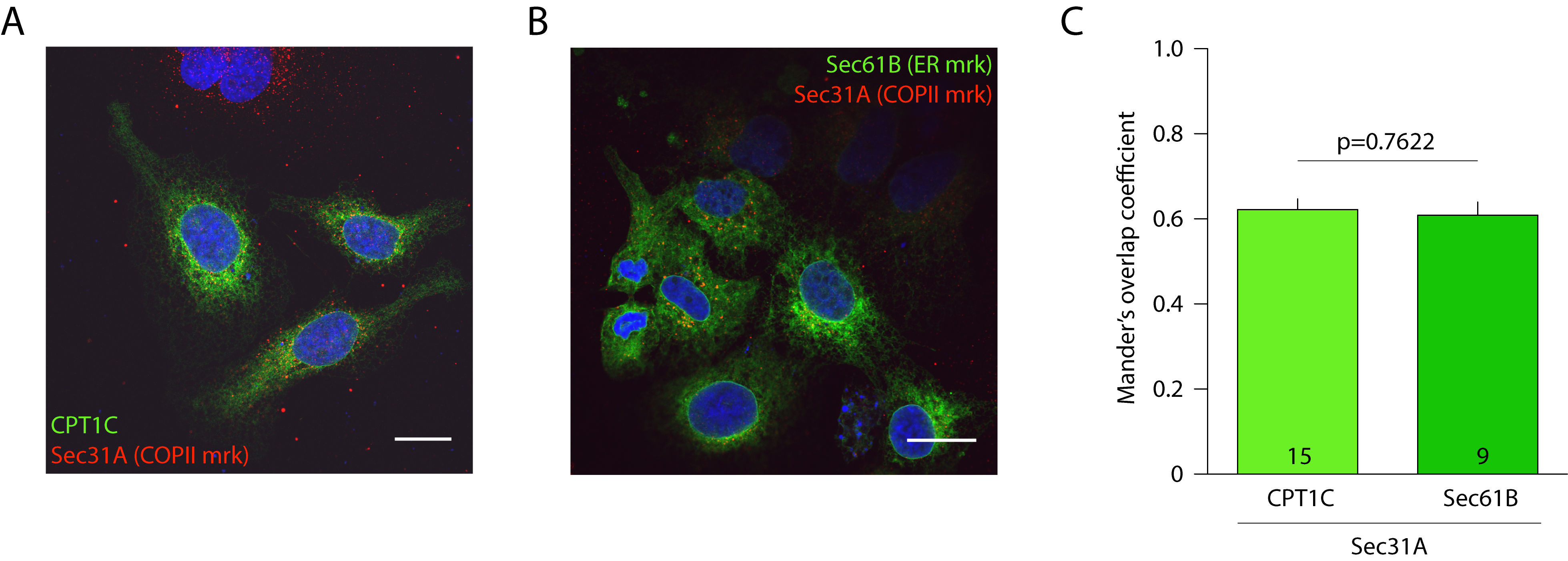

Supplement: Supplementary file 4 [file Image_3.TIF]

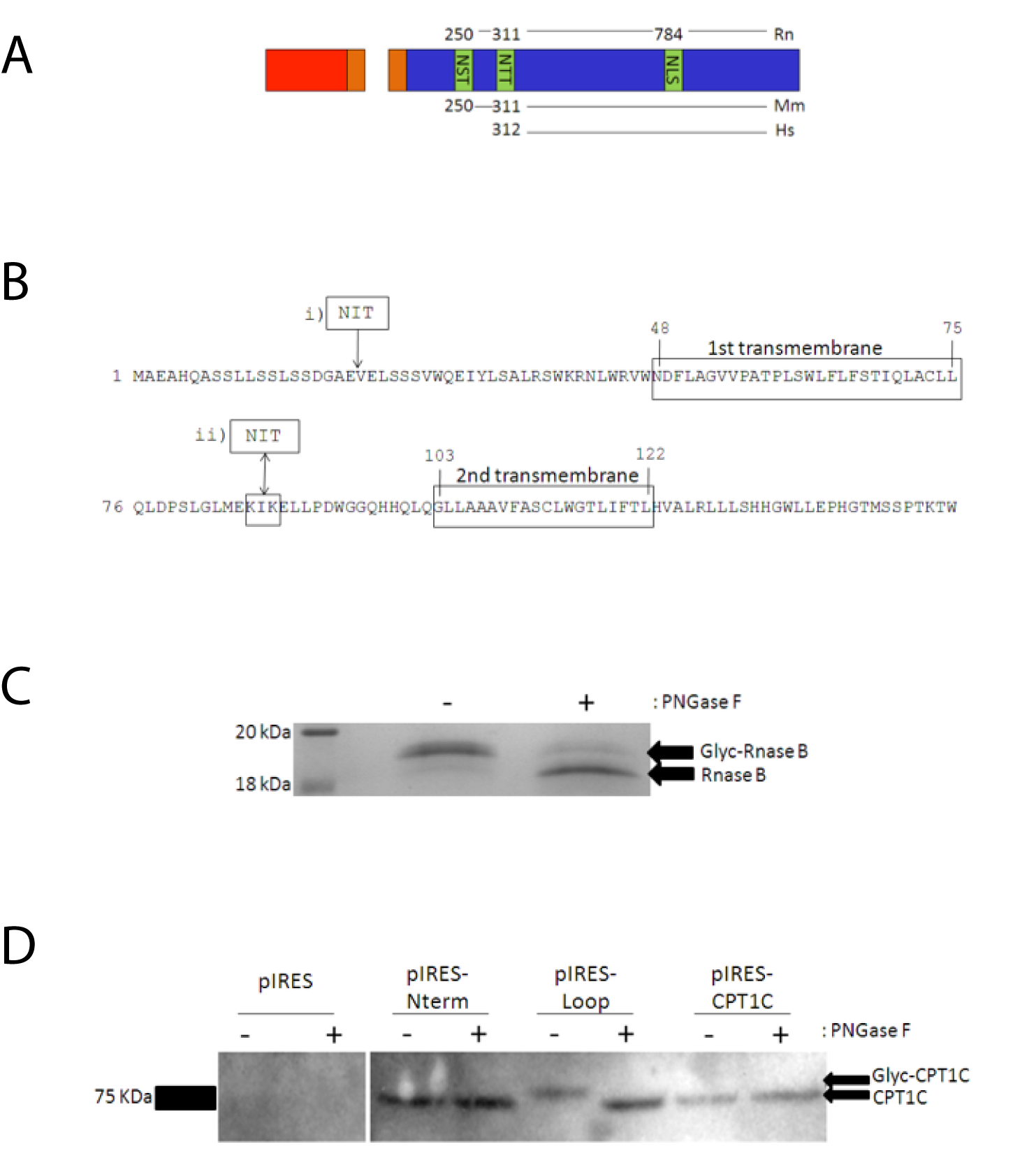

Supplement: Supplementary file 5 [file Image_4.TIF]
